# A reengineered common chain cytokine augments CD8^+^ T cell–dependent immunotherapy

**DOI:** 10.1172/jci.insight.158889

**Published:** 2022-05-23

**Authors:** Anirban Banerjee, Dongge Li, Yizhan Guo, Zhongcheng Mei, Christine Lau, Kelly Chen, John Westwick, Jeffery B. Klauda, Adam Schrum, Eric R. Lazear, Alexander S. Krupnick

**Affiliations:** 1 Department of Surgery, University of Virginia, Charlottesville, Virginia, USA.; 2 Department of Surgery, University of Maryland, Baltimore, Maryland, USA.; 3 Courier Therapeutics, Houston, Texas, USA.; 4 Department of Chemical and Biomolecular Engineering, University of Maryland, College Park, Maryland, USA.; 5 Departments of Molecular Microbiology and Immunology, Surgery, and Biomedical, Biological and Chemical Engineering, University of Missouri, Columbia, Missouri, USA.; 6 Valo Health, Boston, Massachusetts, USA.

**Keywords:** Immunology, Therapeutics, Cytokines, Immunotherapy

## Abstract

Cytokine therapy is limited by undesirable off-target side effects as well as terminal differentiation and exhaustion of chronically stimulated T cells. Here, we describe the signaling properties of a potentially unique cytokine by design, where T cell surface binding and signaling are separated between 2 different families of receptors. This fusion protein cytokine, called OMCPmutIL-2, bound with high affinity to the cytotoxic lymphocyte-defining immunoreceptor NKG2D but signaled through the common γ chain cytokine receptor. In addition to precise activation of cytotoxic T cells due to redirected binding, OMCPmutIL-2 resulted in superior activation of both human and murine CD8^+^ T cells by improving their survival and memory cell generation and decreasing exhaustion. This functional improvement was the direct result of altered signal transduction based on the reorganization of surface membrane lipid rafts that led to Janus kinase-3–mediated phosphorylation of the T cell receptor rather than STAT/AKT signaling intermediates. This potentially novel signaling pathway increased CD8^+^ T cell response to low-affinity antigens, activated nuclear factor of activated T cells transcription factors, and promoted mitochondrial biogenesis. OMCPmutIL-2 thus outperformed other common γ chain cytokines as a catalyst for in vitro CD8^+^ T cell expansion and in vivo CD8^+^ T cell–based immunotherapy.

## Graphical abstract

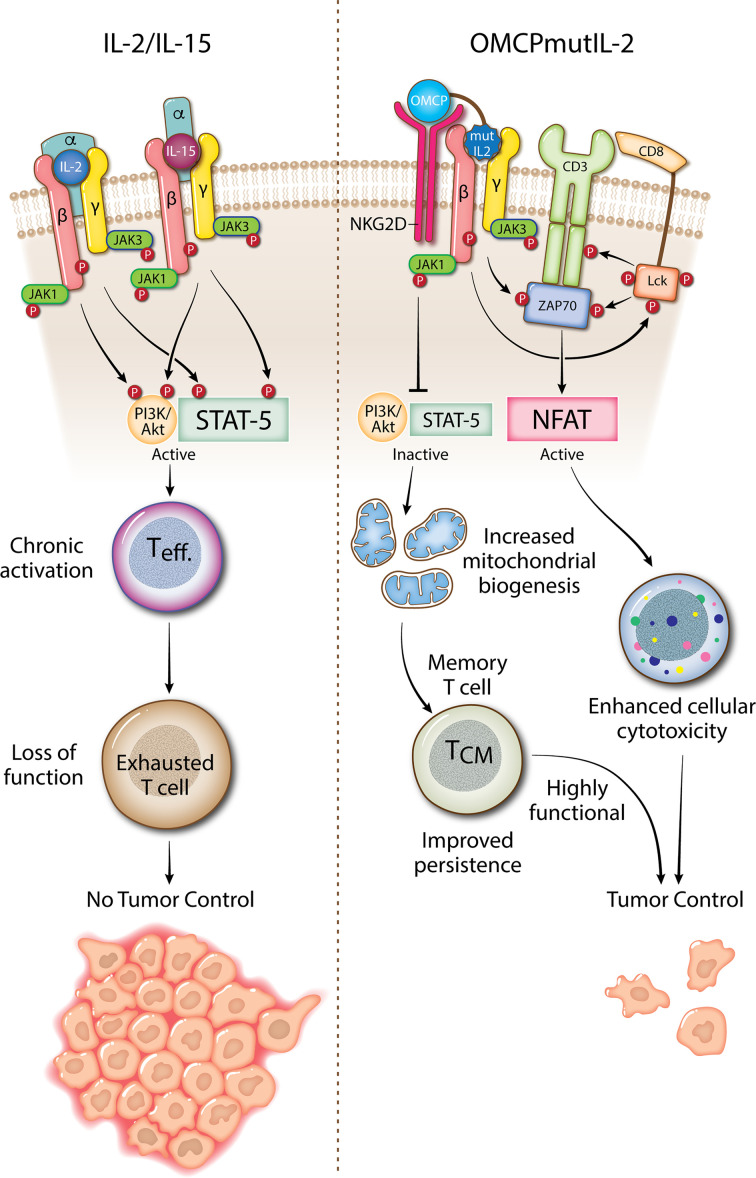

## Introduction

Cytokines are a loosely defined family of small signaling molecules that, while unable to cross the cell membrane, participate in cell signaling and immunomodulation by engaging surface receptors. The common γ chain family constitutes a subset of short-chain 4–helical bundle cytokines, which consist of the interleukins IL-2, IL-4, IL-7, IL-9, IL-15, and IL-21. All cytokines in this family signal through the IL-2 receptor γ chain (CD132) surface receptor (1) and contribute to a very powerful system of immunologic surveillance for infection and altered or foreign tissue. For this reason therapeutic administration of common γ chain cytokines, such as IL-2 and IL-15, has been clinically and experimentally utilized for the treatment of malignancies and infectious disease (2). Administration of the cytokine IL-2, specifically, is considered the first successful human immunotherapy due to its ability to stimulate immunologic clearance of malignancies such as melanoma and renal cell carcinoma in select patients (3, 4). Administration of naturally occurring cytokines such as IL-2, however, must occur in extremely high doses as regulatory T cells express high levels of the CD25 surface receptor for IL-2 (known as the α chain), which facilitates the “capture” of the cytokine at the cell surface. Together with the βγ signaling chains, this forms the trimeric high-affinity αβγ IL-2 receptor on regulatory T cells, which limit the immune response by sequestering IL-2 and other cytokines for autologous use at the expense of effector cell activation. In addition, high-dose cytokine administration leads to systemic toxicity due to “off-target” side effects, such as diffuse capillary leak, pulmonary edema, and cardiomyopathy. This is the direct result of broad expression of both the high-affinity α as well as lower affinity γ chain on a wide variety of tissues, including endothelial cells (5). Prolonged cytokine exposure results in terminal differentiation and exhaustion of T cells, further limiting efficacy (6, 7). Such exhaustion is likely an evolutionary protective mechanism inherent to cytokine signal transduction designed to limit uncontrolled immune activation at sites of inflammation (8).

Attempts to overcome toxicity of cytokine administration have included engineering of multiple cytokine mutants. Since regulatory T cells express high levels of the high-affinity α chain, one strategy to improve cytokine availability to effector cells focuses on limiting α chain binding. This has been attempted by engineering non–α chain–binding muteins (9–12), combining IL-2 with an antibody that blocks α chain interaction (13), or PEGylating IL-2 to both prevent α chain binding and extend serum half-life (14, 15). Alternative strategies, such as extending serum half-life through addition of an Fc chain to cytokines, have been proposed as well (16). Nevertheless, all these strategies have limitations, as broad engagement of multiple cell types and tissues occurs by such engineered cytokines, even in the absence of α chain binding. None of the above-described approaches limit lymphocytes’ exhaustion, as they engage canonical cytokine signaling pathways for cytotoxic lymphocyte activation. The notable failure of PEGylated IL-2 in late-phase clinical trials further supports this notion (17).

Based on the high potential of cytokine therapy and limitations of the strategies described above, we have pioneered an alternative approach to improve both efficacy and safety. To this end, we created a potentially unique cytokine by design where a non–α chain–binding IL-2 mutein, which signals exclusively through the IL-2 receptor βγ chains (mutIL-2), is redirected to bind to cytotoxic lymphocytes using a high-affinity ligand to NKG2D (18). As NKG2D is an immunoreceptor whose expression is restricted solely to cytotoxic lymphocytes, such as NK cells, CD8^+^ T cells, and γδ T cells (19), such redirected binding facilitates immunotherapy by limiting competition from regulatory T cells (20) and eliminating off-target side effects, such as diffuse capillary leak (18). By utilizing a high-affinity virally encoded NKG2D ligand known as orthopoxvirus MHC class I–like protein, or OMCP (21, 22), we take advantage of an evolutionary designed ligand that outperforms antibody-mediated delivery (23).

Nevertheless, the physiology and signaling pathways of this potentially unique cytokine fusion protein, called OMCPmutIL-2, in CD8^+^ T cell activation remained unexplored. Here, we describe that OMCPmutIL-2 activated CD8^+^ T cells by augmenting T cell receptor (TCR) signal transduction rather than activating the canonical STAT/AKT-dependent signaling mechanism shared by other common γ chain cytokines. We demonstrate that this likely unique signaling pattern was the direct result of high-affinity binding of OMCPmutIL-2 to the cell surface, which resulted in the formation of a membrane complex comprising NKG2D, the IL-2 receptor, as well as the TCR within a large lipid raft–like structure. Such membrane reorganization resulted in JAK-mediated phosphorylation of the TCR, rather than STAT5, upon cytokine binding and led to augmented CD8^+^ T cell proliferation, memory formation, and cytotoxicity, as well as decreased activation-induced cell death and exhaustion in an nuclear factor of activated T cell–dependent (NFAT-dependent) manner. Thus, OMCPmutIL-2–expanded CD8^+^ T cells outperformed T cells expanded in IL-2 or IL-15 in multiple murine and human tumor models.

## Results

### OMCPmutIL-2 mediates superior activation of CD8^+^ T cells compared with IL-2 and IL-15.

**Figure 1:**
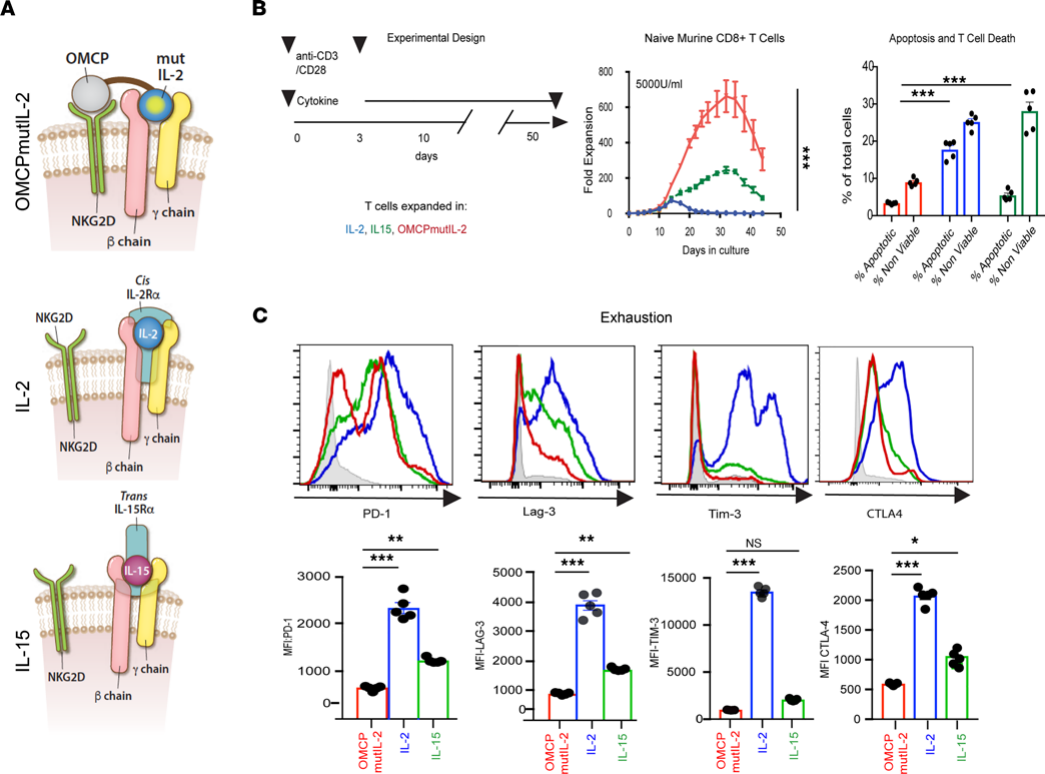
OMCPmutIL-2 mediates superior CD8^+^ T cell expansion accompanied by less exhaustion. (**A**) Schematic rendition of OMCPmutIL-2 binding to cell surface and variability in common γ chain cytokine surface receptor capture, relying on high-affinity α chain in *cis* for IL-2 and in *trans* for IL-15. (**B**) Experimental design and expansion of peripheral blood lymphocyte–derived (PBL-derived) naive CD8^+^CD44^lo^62L^hi^ murine T cells as measured by fold expansion and viability. Apoptotic cells defined as Annexin viability dye^–^ and nonviable cells defined as Annexin viability dye^+^. (**C**) Expression of exhaustion markers on cells expanded in respective cytokines. *P < 0.05; **P < 0.01; ***P < 0.001; *t* test. CTLA-4, cytotoxic T lymphocyte–associated protein 4; Lag-3, lymphocyte activating 3; PD-1, programmed cell death 1.

Naturally occurring common γ chain cytokines, such as IL-2 and IL-15, are captured at the cell surface by their respective γ chains and signal through the βγ chain. OMCPmutIL-2 is a fusion protein that binds with high affinity to NKG2D but signals through the IL-2βγ receptor just like IL-2 and IL-15 (Figure 1A and ref. 18). While NKG2D is an activating receptor, OMCPmutIL-2 does not signal through this receptor (Supplemental Figure 1, A and B; supplemental material available online with this article; https://doi.org/10.1172/jci.insight.158889DS1; and as previously published in ref. 18) but rather limits activation to NKG2D-expressing cytotoxic lymphocytes, such as NK cells, CD8^+^ T cells, and γδ T cells (20), without stimulation of regulatory T cells or vascular endothelium (5, 18). This has led to a previous demonstration that OMCPmutIL-2 provides a distinct advantage for NK cell–mediated immunotherapy in vivo by limiting competition from non-NKG2D-expressing lymphocytes such as CD4^+^ T cells, CD4^+^CD25^+^Foxp3^+^ regulatory T cells, or stromal cells such as vascular endothelium (18).

**Figure 2:**
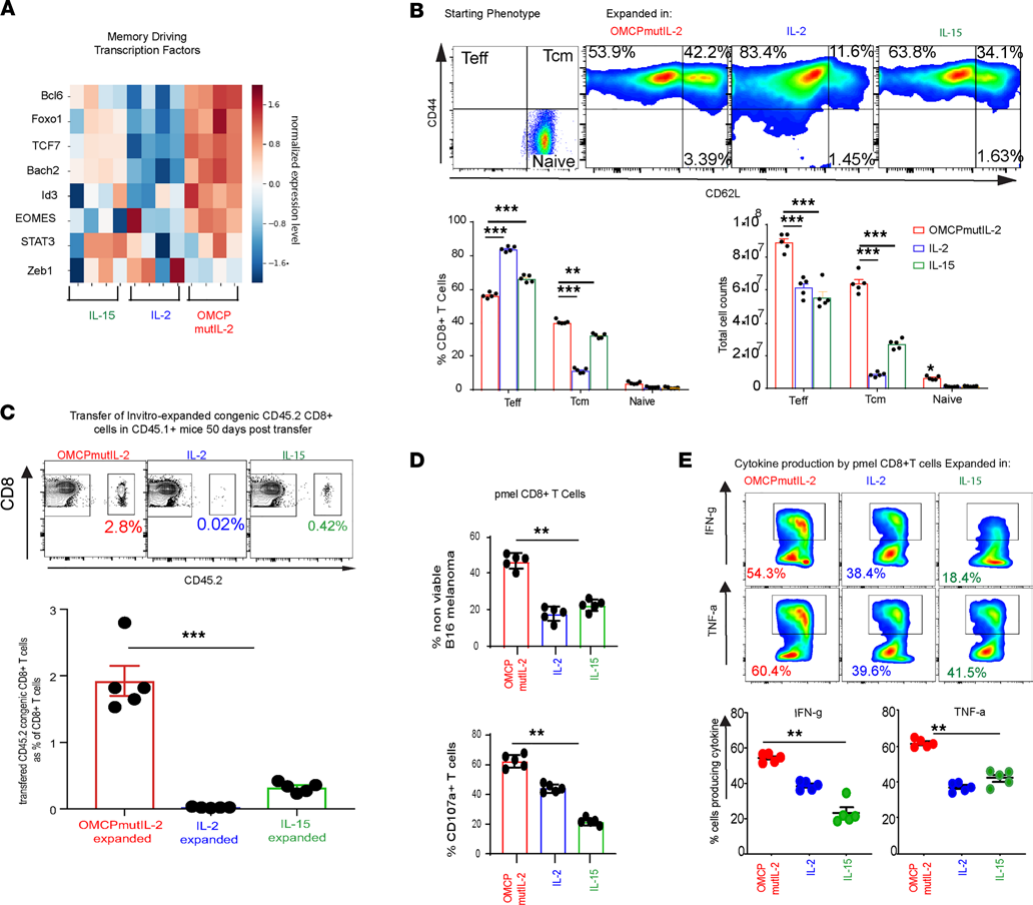
OMCPmutIL-2 mediates CD8^+^ T cell memory generation which shows superior cytotoxic function. (**A**) Heatmap showing relative gene expression of memory-driving transcription factors. (**B**) Relative proportion and number of CD44^lo^62L^hi^ naive, CD44^hi^62L^hi^ central memory (Tcm), or CD44^hi^62L^lo^ effector cells (Teff) after 2 weeks of expansion in IL-2 (blue), IL-15 (green), or OMCPmutIL-2 (red). Representative FACS plots (top), quantitative percentage (bottom left), and total cell count (bottom right) from a starting population of 1 × 10^6^ flow cytometrically sorted naive cells. (**C**) Analysis of spleen 50 days after adoptive transfer of CD45.2 congenic CD8^+^ T cells expanded in wild-type IL-2 (blue), IL-15 (green), or OMCPmutIL-2 (red) prior to transfer. (**D**) In vitro cytotoxicity by murine pmel anti-GP100 TCR-transgenic CD8^+^ T cells as determined by the ability to lyse B16 melanoma (nonviable) and T cell degranulation measured by surface CD107a expression with 1:1 B16/pmel CD8^+^ T cell ratio. (**E**) Effector cytokine (IFN-γ and TNF-α) production by murine pmel anti-GP100 TCR-transgenic CD8^+^ T cells expanded in respective cytokines, upon maximal stimulation. **P < 0.01; ***P < 0.001; *t* test.

CD8^+^ T cells also express NKG2D (24) and can thus bind to and respond to OMCPmutIL-2 in mixed tumor-infiltrating lymphocyte cultures (20). Purified CD8^+^ T cells expanded in OMCPmutIL-2 demonstrated superior proliferation, as well as reduced apoptosis and cell death (Figure 1B), in both murine and human cultures (Supplemental Figure 1, C–E). In addition, OMCPmutIL-2–expanded CD8^+^ T cells demonstrated lower levels of exhaustion markers compared with those expanded in IL-2 or IL-15 (Figure 1C). Gene expression analysis of purified cultures revealed upregulation of memory-driving transcription factors in OMCPmutIL-2 expanded CD8^+^ T cells compared with those expanded in wild-type IL-2 or IL-15 (Figure 2A). When sorted CD8^+^CD44^lo^CD62L^hi^ naive murine T cells were expanded in different cytokines, OMCPmutIL-2 generated a higher proportion of CD8^+^CD44^hi^CD62L^hi^ central memory T cells (Figure 2B). To investigate whether long-lived memory cells were truly generated by such treatment, we transferred 5 × 10^6^ CD45.2^+^ CD8^+^ T cells, expanded for 2 weeks in vitro, into resting CD45.1^+^ congenic mice and noted higher persistence of OMCPmutIL-2–expanded cells in the spleen and peripheral organs 50 days posttransfer (Figure 2C and Supplemental Figure 1F). Taken together these data suggest that OMCPmutIL-2 may initiate a potentially unique “cell-intrinsic” mechanism of activation in CD8^+^ T cells independent of redirected competitive binding. Supporting this notion, purified TCR-transgenic anti-GP100 pmel CD8^+^ T cells (25) expanded in vitro with OMCPmutIL-2 were able to lyse B16 melanoma cells more readily than those expanded in IL-2 or IL-15 (Figure 2D). In addition to increased cytotoxicity, as measured by B16 melanoma cell lysis, CD8^+^ T cells expanded in OMCPmutIL-2 had more potential to produce effector cytokines after maximal stimulation (Figure 2E).

### OMCPmutIL-2 activates CD8^+^ T cells through the NFAT signal transduction pathway rather than STAT5/AKT.

**Figure 3:**
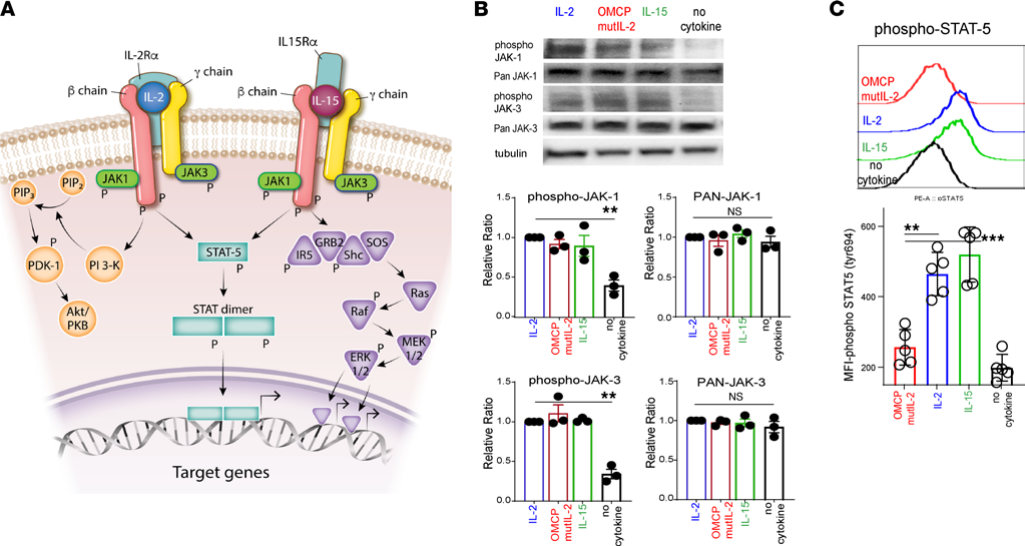
OMCPmutIL-2 facilitates JAK phosphorylation but does not signal through canonical JAK/STAT pathway like IL-2 family of cytokines. (**A**) Canonical signaling pathways described for common γ chain cytokines. (**B**) JAK1 and JAK3 total and phosphorylated levels by Western blot analysis after in vitro activation with anti-CD3/28 stimulation and in the presence of IL-2, IL-15, OMCPmutIL-2, or control (saline). (**C**) Phospho-STAT5 levels in CD8^+^ T cells activated with IL-2, IL-15, OMCPmutIL-2, or saline control in the presence of anti-CD3/28. *P < 0.05; **P < 0.01; ***P < 0.001; *t* test.

**Figure 4:**
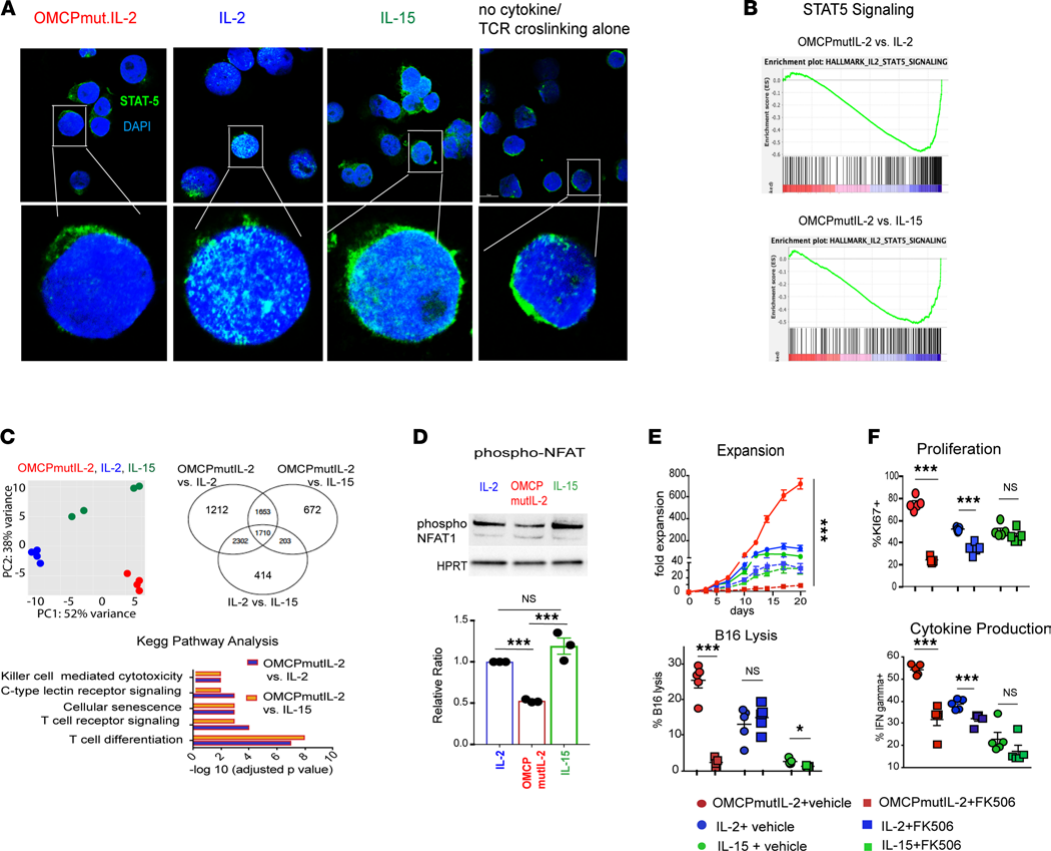
OMCPmutIL-2 facilitates NFAT signaling but does not signal through canonical JAK/STAT pathway like IL-2 family cytokines. (**A**) STAT5 localization by confocal microscopy after in vitro activation with anti-CD3/28 stimulation in the presence of IL-2, IL-15, OMCPmutIL-2, or saline control. Original magnification, 20× (top) and 63× (bottom). (**B**) Gene enrichment analysis for STAT5-dependent genes in memory T cells generated from naive cells in vitro in the presence of IL-2 or IL-15 versus OMCPmutIL-2. (**C**) Principal component analysis between IL-2 (blue), IL-15 (green), and OMCPmutIL-2 (red) and differential gene expression in murine splenic CD8^+^ T cells expanded in IL-2, IL-15, or OMCPmutIL-2 as expressed via Venn diagram and Kyoto Encyclopedia of Genes and Genomes pathway analysis comparing top differential pathways. OMCPmutIL-2 versus IL-2 and OMCPmutIL-2 versus IL-15 are presented in separate graphs (purple for OMCPmutIL-2 vs. IL-2 and orange for OMCPmutIL-2 vs. IL-15). (**D**) Phosphorylated (inactive) NFAT quantification in CD8^+^ T cells after expansion in various cytokines. (**E**) Change in CD8^+^ T cell expansion and cytotoxicity in the presence or absence of NFAT inhibitor FK506. (**F**) Change in CD8^+^ T cell proliferation and cytokine production in the presence or absence of NFAT inhibitor FK506. *P < 0.05; **P < 0.01; ***P < 0.001; *t* test.

To better understand the mechanistic effects of OMCPmutIL-2, we interrogated intermediates known to be a part of the common γ chain cytokine signaling pathway (Figure 3A). After 1 hour of exposing CD8^+^ T cells to plate-bound anti-CD3/CD28 cross-linking antibodies and common γ chain cytokines, we noted similar phosphorylation of JAK1/3 but lower levels of activated phosphorylated STAT5 (phospho-STAT5) and AKT in OMCPmutIL-2–expanded murine and human CD8^+^ T cells compared with those expanded in IL-2 and IL-15 (Figure 3, B and C; and Supplemental Figure 2, A and B). Unlike the case for IL-2 and IL-15 (26), we noted little nuclear STAT5 in CD8^+^ T cells expanded in OMCPmutIL-2 (Figure 4A), and gene expression analysis demonstrated no enrichment for STAT5 signaling gene expression (Figure 4B and Supplemental Figure 2C). Taken together, our data suggested that alternative signaling mechanisms are involved in OMCPmutIL-2–mediated CD8^+^ T cell activation.

Principal component gene expression analysis of CD8^+^ T cells expanded in OMCPmutIL‑2 demonstrated upregulation of multiple pathways affecting cytotoxicity, differentiation, and receptor signaling when compared with CD8^+^ T cells expanded in either IL-2 or IL-15. All such upregulated pathways seemed to share dependency on the NFAT family of transcription factors (Figure 4C). Supporting this notion, lower levels of phosphorylated, inactive NFAT1 were evident in OMCPmutIL-2–expanded T cells (Figure 4D), while total NFAT was similar among all cytokines (Supplemental Figure 2D). Further, gene expression analysis indicated higher expression of transcription factors NFATC1, NFATC2, and NFATC3 in OMCPmutIL-2–expanded CD8^+^ T cells compared with other IL-2 family cytokines (Supplemental Figure 2E). Inhibition of NFAT dephosphorylation using FK506 (27) eliminated proliferation and activation of CD8^+^ T cells by OMCPmutIL-2 but had little effect on CD8^+^ T cells expanded in IL-2 or IL-15 (Figure 4, E and F). This observation suggested that, unlike other members of the common γ chain family of cytokines, CD8^+^ T cell activation by OMCPmutIL-2 is dependent on NFAT-mediated signal transduction.

### Enhanced NFAT signaling mediated by OMCPmutIL-2 is the result of augmented TCR signal transduction.

**Figure 5:**
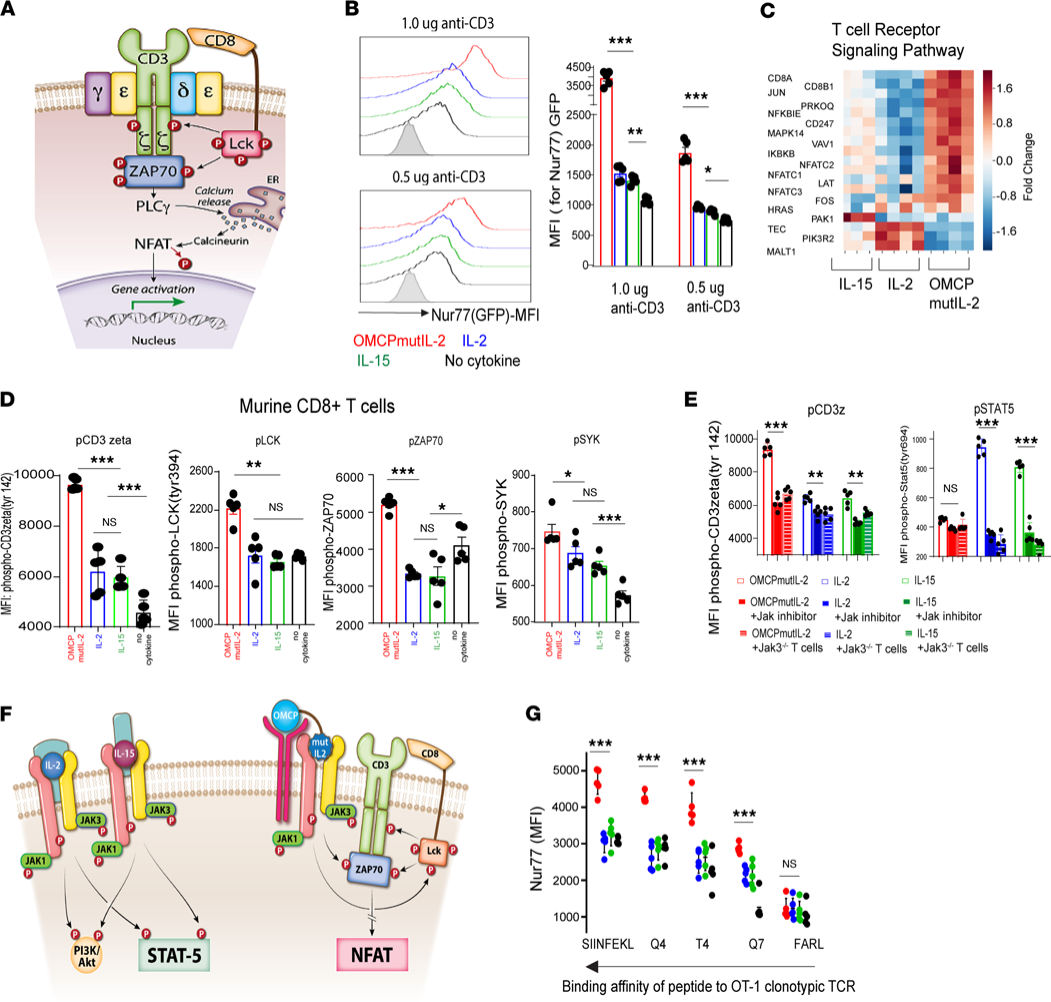
OMCPmutIL-2 promotes NFAT-mediated CD8^+^ T cell activation by augmenting TCR signal transduction. (**A**) Canonical pathway of NFAT activation through TCR signal transduction. (**B**) TCR signaling, as measured by Nur77 upregulation (representative FACS plot, left) in the presence of cytokines and variable concentrations of TCR agonistic antibody (anti-CD3) as well as 2 μg/mL of anti-CD28 (MFI, quantification from 5 individual Nur77GFP mice, right). (**C**) Relative gene expression of the TCR signaling pathway as defined by heatmap analysis in splenic murine CD8^+^ T cells cultured in the presence of anti-CD3/28 and various cytokines. (**D**) Phosphorylation of TCR signaling components of murine CD8^+^ T cells in the presence of wild-type IL-2 (blue), IL-15 (green), or OMCPmutIL-2 (red) or no cytokine (black) with anti-CD3/CD28 cross-linking antibodies. (**E**) Phosphorylation levels of CD3ζ and STAT-5 levels as measured by MFI in murine CD8^+^ T cells in the presence or absence of JAK1/3 inhibitor as compared with CD8^+^ T cells derived from Jak3^–/–^ mice. (**F**) Graphical representation of OMCPmutIL-2–mediated activation of NFAT activation at the expense of the canonical STAT5/AKT signaling pathways. (**G**) TCR signal transduction, as measured by Nur77 expression, in OT-1 clonotypic CD8^+^ T cells incubated with dendritic cells loaded with various SIINFEKL mutants with variable TCR binding avidity. *P < 0.05; **P < 0.01; ***P < 0.001; *t* test.

NFAT activation is mediated by TCR-initiated calcium flux (ref. 28 and Figure 5A). In the presence of TCR stimulation, OMCPmutIL-2 mediated robust signal transduction, as measured by upregulation of Nur77 (ref. 29 and Figure 5B) and gene expression analysis of TCR-specific genes (Figure 5C). No such signaling was evident in the absence of TCR ligation (Supplemental Figure 3A). Interestingly, TCR signal transduction was augmented by IL-2 and IL-15 as well, albeit much less than that evident in the presence of OMCPmutIL-2 (Figure 5B). These data suggest that OMCPmutIL-2 may augment a signaling pathway that is operational in other common γ chain cytokines as well (30).

To study this in more detail, we next evaluated phosphorylation of the TCR after cytokine exposure. In the absence of TCR cross-linking, exposure of CD8^+^ T cells to IL-2, IL-15, or OMCPmutIL-2 resulted in detectable, but equivalent, increase in phosphorylation of components of the TCR (Supplemental Figure 3B). However, in the presence of anti-CD3/28 cross-linking antibodies, OMCPmutIL-2 augmented phosphorylation of multiple components of the TCR, including proximal cell surface components such as LCK and CD3ζ as well as intracellular signaling intermediates such as Zap70 and Syk, to a much greater degree than IL-2 or IL-15 in both murine and human CD8^+^ T cells (Figure 5D and Supplemental Figure 3C). Both IL-2 and IL-15 augmented phosphorylation of some (such as CD3ζ) but not all components of the TCR and to a much lesser extent than that of OMCPmutIL-2 (Figure 5D and Supplemental Figure 3C). This suggested that kinases normally associated with the IL-2 signal transduction pathway may contribute to the phosphorylation of the TCR and this effect is substantially enhanced by OMCPmutIL-2. Consistent with these data, chemical or genetic inhibition of JAK family signaling intermediates eliminated STAT5 phosphorylation in CD8^+^ T cells cultured with IL-2 and IL-15 and decreased phosphorylation of the TCR in OMCPmutIL-2–cultured cells (Figure 5E and Supplemental Figure 3, D and E). Collectively, these observations suggest that OMCPmutIL-2 accentuated the naturally occurring JAK-mediated facilitation of TCR signal transduction, resulting in NFAT activation at the expense of the STAT5/AKT signaling pathways (Figure 5F).

We next considered the possibility that such activation of the TCR by OMCPmutIL-2 may improve functional recognition of poorly immunogenic antigens. Indeed, OMCPmutIL-2 augmented TCR signaling when exposed to mutants of the ovalbumin peptide SIINFEKL with lower binding affinity (ref. 31, Figure 5G, and Supplemental Figure 3F). Thus, OMCPmutIL-2, while unable to directly signal through the TCR in and of itself, may alter TCR signaling to convert weak peptide interactions to model those of high-affinity binding and signaling.

### Organization of lipid raft complexes by OMCPmutIL-2 mediates phosphorylation of the TCR.

**Figure 6:**
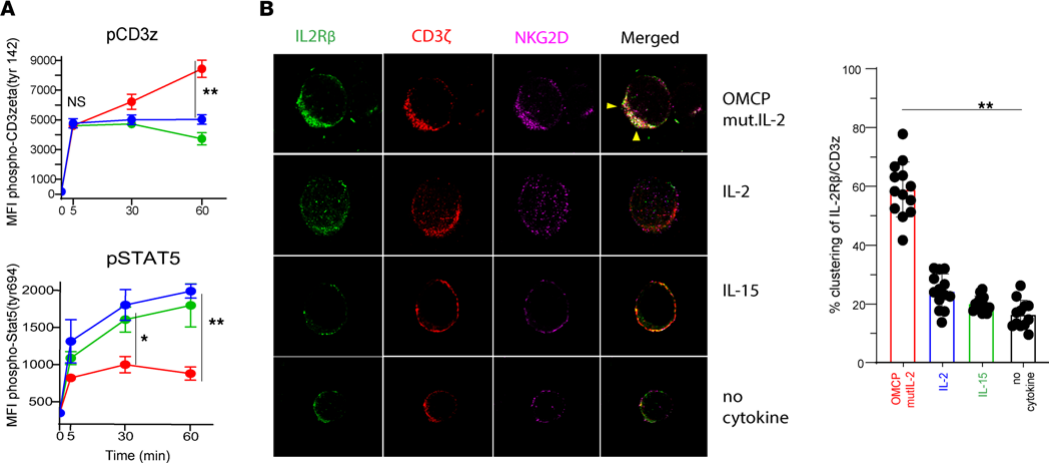
OMCPmutIL-2 facilitates TCR phosphorylation through the reorganization of surface membrane receptors. (**A**) Time course analysis (0–60 minutes) of phosphorylation of CD3ζ and STAT5 in murine CD8^+^ T cells stimulated with OMCPmutIL-2 (red), wild-type IL-2 (blue), and IL-15 (green). (**B**) (Left) High-resolution confocal microscopy of human CD8^+^ T cell surface receptors IL-2Rβ (CD122) (green), CD3ζ (red), and NKG2D (pink) after 1 hour in the presence of plate-bound anti-CD3/28 with different cytokines. Visible colocalization of receptors is shown by arrows (yellow). Original magnification, ×63. (Right) Percentage of T cells demonstrating visible clustering of CD3ζ and IL-2Rβ per high-power field. *n* = 13. *P < 0.05; **P < 0.01; ***P < 0.001; *t* test.

**Figure 7:**
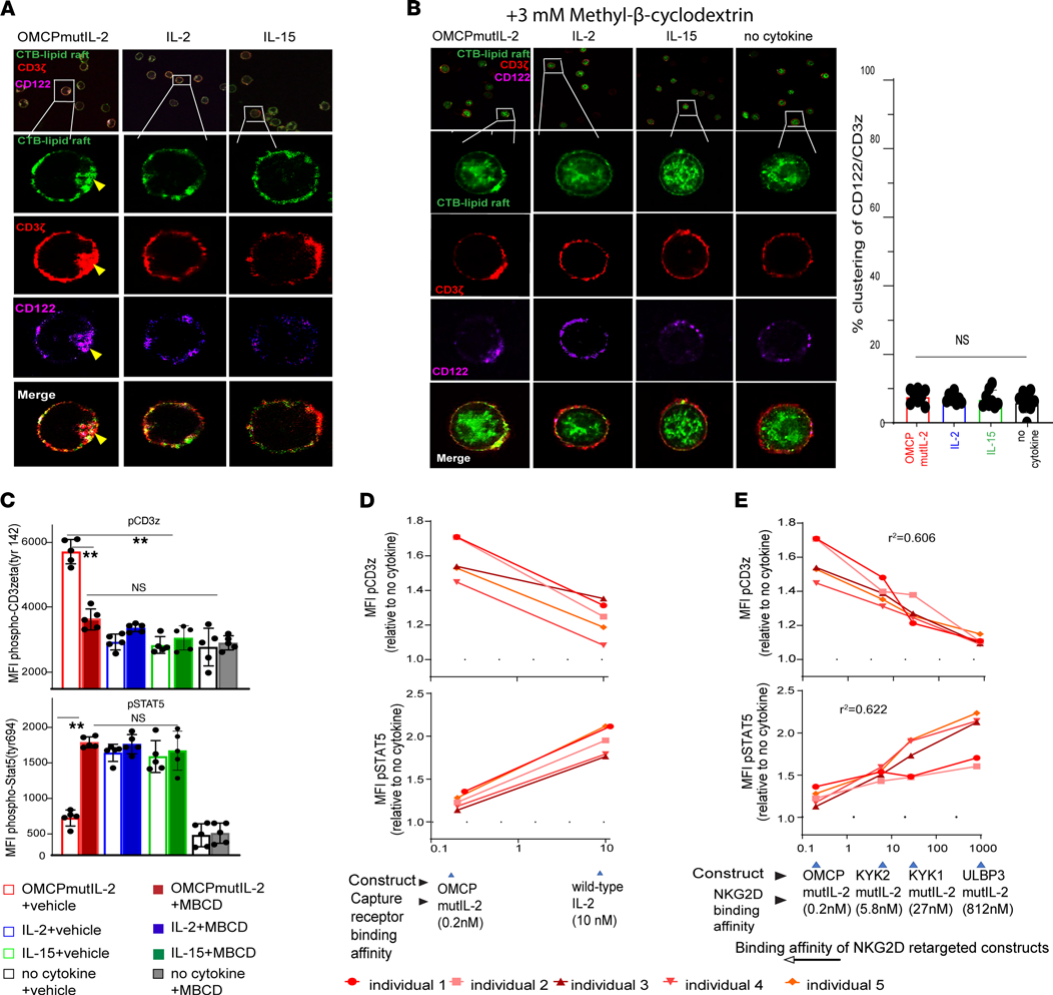
OMCPmutIL-2–mediated reorganization of surface membrane receptors is lipid raft dependent, and downstream TCR phosphorylation depends on cytokine binding affinity. (**A**) High-resolution confocal microscopy of human CD8^+^ T cells evaluating receptor localization/clustering of CD3ζ (red) and IL-2Rβ/CD122 (pink) with lipid rafts (stained by Cholera Toxin B, CTB, in green). Clustering of receptors is shown by yellow arrow. (**B**) High-resolution confocal microscopy of human CD8^+^ T cell surface receptors (left panel) IL-2Rβ (CD122) (green), CD3ζ (red), and lipid rafts (stained by CTB in green) after lipid raft disruption by Methyl-β-cyclodextrin (MBCD) demonstrates no visible colocalization of receptors. Percentage clustering (right panel) shows little clustering and no difference between groups after MBCD treatment. Original magnification, ×63. (**C**) Flow cytometric analysis of CD3ζ and STAT5 phosphorylation after 1 hour of stimulation in various cytokines in the presence or absence of MBCD. (**D**) Phospho-CD3ζ and phospho-STAT5 expression in human PBL-derived CD8^+^ T cells after culture with OMCPmutIL-2 versus IL-2. (**E**) Phospho-CD3ζ and phospho-STAT5 expression in human PBL-derived CD8^+^ T cells after culture with mutIL-2–redirected constructs with different NKG2D ligands having different binding affinity. **P < 0.01; *t* test (**B**–**D**) or ANOVA (**E**).

To better understand JAK-mediated phosphorylation of the TCR, we evaluated multiple time points and noted that CD3ζ phosphorylation was identical between all cytokines 5 minutes after activation but began to diverge after 30 minutes (Figure 6A). This suggested that temporal events play a major role in signaling properties of OMCPmutIL-2. As lipid raft microdomains rich in sphingomyelin and cholesterol are involved in both IL-2 receptor and TCR signaling (32, 33), we next evaluated membrane organization in cytokine-exposed human CD8^+^ T cells. We noted that after 1 hour of exposure to OMCPmutIL-2, the IL-2 receptor, NKG2D, and the TCR (CD3ζ) colocalized into 1 large structure (Figure 6B) enriched for lipid raft–defining ganglioside GM1 as defined by Cholera Toxin B (CTB) labeling (Figure 7A). While we did not find it surprising that NKG2D and IL-2 receptor colocalized in the presence of OMCPmutIL-2 based on its design as a dual receptor binder, the finding that the TCR was included in such macro-complexes was unexpected. Taken together with the CTB ganglioside GM1 labeling, this suggested that OMCPmutIL-2 mediated the formation of a potentially unique lipid raft–like macro-complex that allowed clustering of the TCR with components of the IL-2 receptor. Disruption of lipid rafts using 3 mM Methyl-β-cyclodextrin (MBCD; ref. 34) reduced such macro-complex formation (Figure 7B) and TCR phosphorylation by OMCPmutIL-2 (Figure 7C). Taken together these data suggest that OMCPmutIL-2 may improve colocalization of the TCR and IL-2 receptor due to alteration of lipid raft biology.

It has been previously demonstrated that the size transition from micro- to macro-structured lipid domains, such as the structures evident in OMCPmutIL-2–activated T cells, depends on the line tension between the ordered raft domains and the disordered domains (35). Such tension may be increased by ligands’ binding to raft-associated receptors and modulation of protein-lipid interaction (36, 37). As described in the Methods section, we modeled domain size using the model presented by Usery et al. (35) with parameters associated with leaflet dipole distances and lipid areas that conform to raft domains with sphingolipids (38) and GM1 (39). The raft electrostatic energy controlled by the lipid dipoles plus the line tension energy was used to find the minimum energy based on the domain size (Supplemental Figure 4A). Considering typical line tension values, the domain size exponentially increased with a linear increase in line tension (Supplemental Figure 4B). Since the binding affinity of OMCP for NKG2D was substantially higher than that of wild-type IL-2 to the IL-2 receptor α chain (Figure 7D), we hypothesized that binding affinity may affect the formation of macro lipid domains and phosphorylation of the TCR. To this end we next varied the binding domain from the high-affinity OMCP to other moieties with lower affinity for NKG2D based on alternative ligands or single chain fragments of anti-NKG2D antibodies (40). Stimulation of purified human CD8^+^ T cells with such constructs demonstrated a linear correlation between binding affinity and TCR phosphorylation and a negative relationship to STAT5 phosphorylation (Figure 7E) with no organization of lipid macrodomains (Supplemental Figure 4C). Such data suggest that surface binding affinity may directly influence cytokine signaling properties.

### Absence of prolonged AKT activation by OMCPmutIL-2 contributes to mitochondria-mediated memory generation and cellular fitness.

While it is described that activation of NFAT contributes to augmented cytotoxicity and proliferation of T cells, it has not previously been linked to memory T cell generation to our knowledge (27). In fact, in an almost counterintuitive finding, inhibition of NFAT with FK506 improved the viability and expansion of CD8^+^CD44^hi^CD62L^hi^ central memory T cells after cytokine stimulation (Supplemental Figure 5, A and B). Thus, the relevant signaling mechanisms linking OMCPmutIL-2–mediated NFAT activation to the generation of Tcm remained unclear to us.

**Figure 8:**
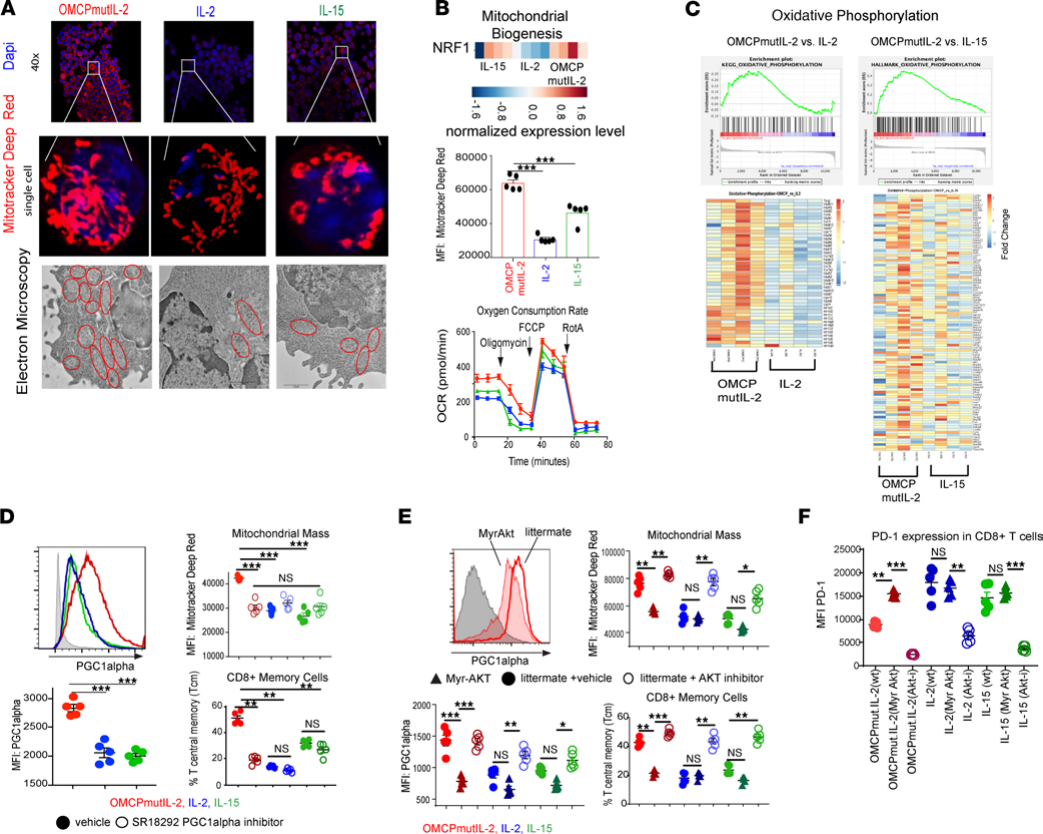
OMCPmutIL-2 facilitates mitochondrial biogenesis by avoiding chronic AKT activation. (**A**) Quantification of mitochondria using high-resolution confocal microscopy using MitoTracker Deep Red dye (upper 2 panels) and electron microscopy (lower panel) depicting increased number of fused mitochondria in OMCPmutIL-2–treated CD8^+^ T cells. Original magnification, ×63 (middle panel) and ×1000 (lower panel). (**B**) Gene expression analysis showing increased NRF1 transcription factors in OMCPmutIL-2–expanded CD8^+^ T cells (upper panel). Flow cytometric analysis of mitochondrial membrane potential (middle) and oxygen consumption rate by Seahorse assay (lower) in murine CD8^+^ T cells expanded in IL-2 (blue), IL-15 (green), or OMCPmutIL-2 (red). (**C**) Analysis of differential expression of genes involved in oxidative phosphorylation as expressed by enrichment scores and heatmaps. (**D**) Flow cytometric quantification of PGC-1α and mitochondrial mass and Tcm after inhibition of PGC-1α. (**E**) PGC-1α levels, mitochondrial mass, and relative proportion of Tcm in the presence of constitutively active AKT (myrAKT mice) or pharmacologic AKT inhibition. (**F**) PD-1 expression as measured by MFI of murine CD8^+^ T cells in the presence of constitutively active AKT (myrAKT) or AKT inhibition. *P < 0.05; **P < 0.01; ***P < 0.001; *t* test.

It has been reported that metabolic reprograming and mitochondrial biogenesis can control CD8^+^ T cell memory formation, viability, and overall fitness (41, 42). When we tested this, we noted a higher density of fused mitochondria, as measured by mitochondria deep red staining as well as electron microscopy in OMCPmutIL-2–expanded CD8^+^ T cells (Figure 8A). This was accompanied by improved mitochondrial function as evidenced by an increase in mitochondrial mass, increased baseline oxygen consumption, and greater mitochondrial membrane potential (Figure 8B and Supplemental Figure 5C). Gene expression analysis showed a metabolic reliance on oxidative phosphorylation rather than glycolysis (Figure 8C and Supplemental Figure 5D). As we found increased expression of nuclear respiratory factor 1 (NRF1) (Figure 8B) and anticipated increased mitochondrial biogenesis, we evaluated peroxisome proliferator–activated receptor γ coactivator 1α (PGC-1α) because NRF1 is a key downstream transcription factor of PGC-1α. We noted upregulation of PGC-1α, a nodal regulator of mitochondrial biogenesis (43), in OMCPmutIL-2–expanded T cells (Figure 8D). To evaluate whether PGC-1α upregulation contributed to OMCPmutIL-2 physiology, we tested chemical PGC-1α inhibitor SR18292 (44) and noted a decrease in mitochondrial mass and Tcm expansion and viability (Figure 8D and Supplemental Figure 5E). These data suggested a link between OMCPmutIL-2, PGC-1α–mediated mitochondrial biogenesis, CD8^+^ T cell metabolic fitness, and memory cell generation.

It has been demonstrated that chronic AKT stimulation leads to loss of active PGC-1α and mitochondrial dysfunction (45). As common γ chain cytokines lead to robust activation of the PI3K/AKT pathway, we asked whether the noncanonical NFAT-mediated signaling pathways may perhaps facilitate PGC-1α upregulation due to decreased activation of AKT. In support of this notion, chemical inhibition of AKT during CD8^+^ T cell expansion increased PGC-1α, mitochondrial mass, and Tcm generation. On the contrary, CD8^+^ T cells from myristoylated AKT mice (myrAKT mice), which have a constitutively active form of AKT (46), failed to upregulate mitochondria content or form Tcm when expanded in OMCPmutIL-2, with no increase in proliferation or viability (Figure 8E and Supplemental Figure 5F). In addition, OMCPmutIL-2–expanded CD8^+^ T cells from myrAKT mice demonstrated higher levels of exhaustion compared with wild-type littermate controls (Figure 8F). Thus, improved mitochondrial biogenesis could be linked to amelioration of T cell exhaustion. Taken together our data suggest that OMCPmutIL-2 facilitates CD8^+^ T cell proliferation and cytotoxicity directly due to NFAT activation but improves memory formation, viability, and exhaustion indirectly through removing the block on mitochondrial biogenesis induced by chronic PI3K/AKT activation.

### OMCPmutIL-2 improves T cell–mediated immunotherapy.

**Figure 9:**
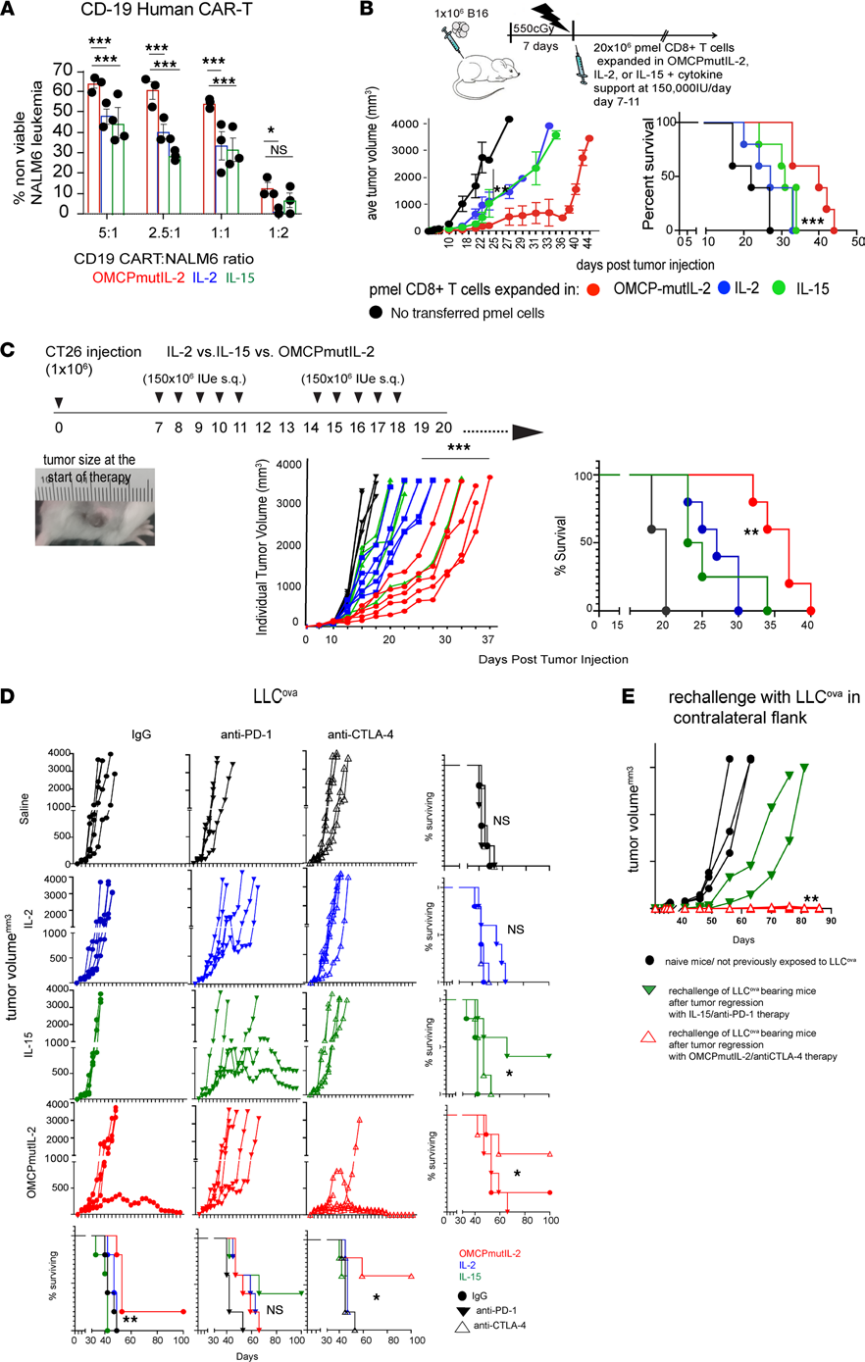
Increased antitumor activity of OMCPmutIL-2–expanded CD8^+^ T cells provides significant improvement in tumor immunotherapy. (**A**) In vitro cytotoxicity of anti-CD19 CAR T cells, expanded in various cytokines, against NALM6 CD19-expressing tumors. (**B**) Experimental design, tumor growth, and survival of B16 melanoma–bearing mice treated with adoptive transfer of pmel anti-GP100 TCR-transgenic CD8^+^ T cells expanded in the presence of IL-2 (blue), IL-15 (green), or OMCPmutIL-2 (red). (**C**) Experimental design, tumor growth, and survival of mice bearing CT26 colon cancer tumors. (**D**) Tumor growth and survival percentage of mice bearing tumor antigen ovalbumin expressing Lewis lung carcinoma (LLC^ova^) in combinatorial immunotherapy with cytokines and checkpoint inhibitors CTLA-4 and PD-1. (**E**) Tumor growth in surviving mice after rechallenge with LLC^ova^. *P < 0.05; **P < 0.01; ***P < 0.001; *t* test. s.q., subcutaneous.

To directly study the physiology described above in tumor immunotherapy, we next generated human anti-CD19 chimeric antigen receptor (CAR) T cells in IL-2, IL-15, or OMCPmutIL-2 and noted improved lysis of human tumors in vitro (Figure 9A). To extend this to an in vivo model of adoptive transfer immunotherapy, we next expanded purified TCR-transgenic GP100-reactive CD8^+^ T cells (47) in IL-2, IL-15, or OMCPmutIL-2 for 2 weeks and adoptively transferred them to C57BL/6 mice bearing established B16 melanoma. Superior tumor control and improved survival were evident in mice treated with OMCPmutIL-2–expanded CD8^+^ T cells compared with those expanded by either IL-2 or IL-15 (Figure 9B).

Based on these data, we next tested OMCPmutIL-2 as a reagent for in vivo immunotherapy in the absence of T cell adoptive transfer. While we have previously defined NK-based immunotherapy potential of OMCPmutIL-2 using NK-responsive tumor cell lines, the CT26 colon cancer cell line is responsive to CD8^+^ T cells (48). Indeed, growth and survival of mice implanted with CT26 were substantially improved when treated with once-a-day injection of subcutaneous OMCPmutIL-2 compared with IL-2 or IL-15 (Figure 9C). Lewis lung carcinoma (LLC), a spontaneously arising and highly aggressive C57BL/6 lung cancer adenocarcinoma, is primarily responsive to NK cells (18, 49). However, transduction with the immunodominant antigen ovalbumin (LLC^ova^) allows for evaluation and modification of T cell–mediated responses. Once-a-day subcutaneous injection of OMCPmutIL-2 ameliorated the tumor growth of mice bearing established LLC^ova^ tumors and increased survival (Figure 9D). OMCPmutIL-2 also augmented the number of circulating antigen-specific CD8^+^ T cells 2 weeks after the start of treatment (Supplemental Figure 6A). When combined with checkpoint inhibition, OMCPmutIL-2 substantially improved survival of mice that received concomitant anti–CTLA-4, resulting in tumor regression in 3 out of 5 mice, while PD-1 blockade potentiated IL-15–based immunotherapy (Figure 9D). In addition, unlike the case for IL-15/PD-1 blockade, mice treated with OMCPmutIL-2 in combination with anti–CTLA-4 remained resistant to tumor rechallenge without additional treatment, suggesting long-term immunity (Figure 9E). Administration of OMCPmutIL-2 did not result in the formation of antidrug antibodies in mice or predicted human immunogenicity using in silico modeling (Supplemental Figure 6B). Taken together with our previously published data (18, 20), such results suggest that OMCPmutIL-2 represents a reagent able to activate both innate and adaptive arms of the immune response for favorable control of malignancies.

## Discussion

Despite their structural similarities and shared receptor use, different common γ chain cytokines mediate different immunologic responses in target cells (41, 50). This variability has been ascribed to context-dependent expression of high-affinity “capture receptors” on various cell types as well as reliance on different STAT family members for signal transduction (1). However, potential “noncanonical” signaling pathways have been poorly defined. Specifically, the concept that common γ chain cytokines can influence the threshold for TCR signaling has been suggested but never fully explored. Au-Yeung and colleagues, for example, demonstrated that both IL-2 and IL-15 can reduce the threshold for TCR signaling in CD8^+^ T cells but acknowledge limited mechanistic insight into the etiology this phenomenon (30). Others have described crosstalk between the IL-2 receptor and the TCR, suggesting a bidirectional interaction between these 2 complex signal transduction pathways without mechanistic details (51). It has similarly been suggested that JAK inhibitors may be used clinically to blunt TCR responses in transplant-mediated rejection, despite limited mechanism to explain this phenomenon (52).

Here we demonstrate that a potentially novel structure of an engineered redirected common γ chain cytokine predominantly functions through JAK-mediated phosphorylation of the TCR due to structural reorganization of ordered membrane domains into large complexes. While it has been suggested that clustering of small lipid rafts into larger microdomains and macrodomains can bring together signaling components that rarely associate under steady-state conditions (53–55), approaches to prove this phenomenon have been poorly defined. In this work, we demonstrate both experimentally and through predictive modeling that unique properties of OMCPmutIL-2, particularly the high-affinity binding to NKG2D and the low-affinity interaction with the IL-2 receptor, allow for aggregation of IL-2 receptor and the TCR (32). While we recognize that OMCP, like all NKG2D ligands, is an MHC class I mimotope (19), we did not detect TCR signal transduction in cells exposed to OMCPmutIL-2 in the absence of concomitant TCR stimulation (Supplemental Figure 3, A and B). Rather we demonstrate that aggregation of TCR along with the IL-2 receptor leads to a signaling entity that accentuates a unique aspect of “redirected” JAK signal transduction. This mechanism of action is thus substantially different from the recently described H9T IL-2 mutant, which alters signaling and metabolic reprogramming of T cells (56), but supports the notion that reduced STAT5 signaling rescues CD8^+^ T cells from exhaustion and altered signaling capacity with maintenance of stem cell–like memory potential may be beneficial for immunotherapy.

Among all naturally occurring NKG2D ligands, OMCP is ideally suited for delivery of cytokines, or other immunomodulators, due to its extremely high binding affinity to cytotoxic lymphocytes. Additionally, OMCP is produced at upward of 3000 copies per cell per second and secreted from the cytosol of mammalian cells (21). Such properties substantially facilitate manufacturing of OMCP fusion proteins from Chinese hamster ovary (CHO) cells. As OMCP binds to NKG2D as a single domain, it provides advantages over antibody-mediated delivery, where dimerization of heavy and light chains must occur for optimal target binding. In addition, OMCP’s small size facilitates tissue penetration over larger antibodies (57). Furthermore, monomeric OMCP binding does not result in NKG2D costimulation (Supplemental Figure 1A), a phenomenon that might occur with antibody-mediated clustering of receptors. Thus, OMCP-mediated targeting of cytotoxic lymphocytes provides a clear and defined method of activation that relies solely on the activating moiety linked to OMCP. Furthermore, as the tumor microenvironment limits TCR activation, due to direct disruption of TCR signal transduction (58) or the general low avidity of tumor-associated self-antigens (59), OMCPmutIL-2 represents a unique immunostimulatory agent that can augment CD8^+^ T cell recognition of antigens.

PD-1 decreases signaling through CD28 and TCR by dephosphorylating intracellular complexes (60, 61). Thus PD-1 blockade functions primarily to limit CD8^+^ T cell exhaustion in a “cell-intrinsic” manner. On the other hand, CTLA-4 downregulates T cell activation through competitive interaction with CD80 and CD86. Thus CTLA-4 blockade affects multiple “cell-intrinsic” and “cell-extrinsic” aspects of the immune response, including inactivation of regulatory T cells and alteration of dendritic cell antigen presentation (62). As we described limited exhaustion of OMCPmutIL-2–expanded T cells, we anticipated that PD-1 blockade would offer limited therapeutic advantage over cytokine alone. Along the same lines anti–CTLA-4 therapy could have a synergistic benefit, which was indeed the case. Such data further define the power of rational combination therapy based on understanding mechanism/s of action.

The finding that superior T cell survival and memory formation occurred due to mitochondrial biogenesis in OMCPmutIL-2–expanded T cells further strengthens the link between mitochondrial biology and T cell fitness (41). It is especially interesting that mitochondrial biogenesis in activated T cells was the result of disinhibition of chronic AKT activation rather than an active process induced by OMCPmutIL-2. Inhibition of mitochondrial generation secondary to prolonged activation of cytokine-related signaling is likely an evolutionary protective mechanism designed to prevent exuberant immune responses at sites of infection (45). By avoiding such canonical signaling pathways, OMCPmutIL-2 bypassed these regulatory mechanisms, leading to unexpected, yet favorable properties of mitochondrially mediated fitness. Thus, CD8^+^ T cells generated by OMCPmutIL-2, despite having a memory-like phenotype, maintained cytotoxic properties by avoiding functional exhaustion. Our data for the first time highlight a potentially novel interaction between TCR and IL-2 receptor subunits shaping downstream signaling pathways and creating improved CD8^+^ T cells, which can be utilized in CAR T cell therapy as well as adoptive transfer therapy to achieve better clinical outcomes. Taken together our data point to the utility of designing novel cytokines that tap into unique signaling pathways to improve immunotherapy.

## Methods

### Animals

All mice used in this study were male and were between 8 and 12 weeks old. We purchased C57BL/6; B6.Cg-*Thy1^a^*/Cy Tg (TcraTcrb)8Rest/J (Pmel); B6.SJL-*Ptprc^a^ Pepc^b^*/BoyJ (B6 CD45.1); *Jak3^–/–^* (strain 002852); Nur77^GFP^ and C57BL/6-Tg (TcraTcrb)1100Mjb/J (OT-1) from The Jackson Laboratory. MyrAkt mice with a constitutive Akt activation carrying the transgene was generated by backcrossing onto C57BL/6 background. About 50 % of the offspring carried the transgene, which was identified by genomic PCR screening using the following primers: PKB-forward: 5′ TGACACCAGGTATTTTGATGA 3′ and PKB-reverse: 5′ TGTTGGACCCAGCTTTGCAG 3′. Animals were housed in a barrier facility in air-filtered cages and allowed free access to food and water.

### Cytokines

Both wild-type human IL-2 and OMCPmutIL-2 were produced through transient transfection in CHO cell line (Celltheon) based on previously described methods (18). Briefly, CHO cells were transfected with an OMCPmutIL-2 expression plasmid. Since OMCPmutIL-2 is secreted into the media, the cells were grown in suspension culture, and the supernatant was harvested at a defined period (2 weeks) when the cell viability was more than 70%. OMCPmutIL-2–containing supernatant was then clarified and buffer exchanged before capture and purification on Ni-NTA columns. The eluted protein was then buffer exchanged into PBS and flash-frozen until use. IL-15 and IL-2 were obtained from the NIH cytokine repository. To standardize comparison of wild-type IL-2 and OMCPmutIL-2, both cytokines were dosed on equimolar basis. Wild-type IL-2 has a specific activity of 15 × 10^6^ U/mg (63). Thus, based on the molecular weight of 15.5 kDa, a 4.4 μM solution was equivalent to 1000 U/μL. Both IL-2 and OMCPmutIL-2 were dosed on an equimolar basis based on units of activity of IL-2. Equimolar dose of IL-15 was calculated based on specific activity of IL-15 provided by NIH biorepository. As described in our previous work (18), the IL-2 mutein used for construction of OMCPmutIL-2 is based on the human cytokine due to its ability to cross-react with and activate murine lymphocytes (64).

### Flow cytometry

All flow cytometric analysis was performed using saturating concentrations of fluorochrome-conjugated antibodies. All antibodies were purchased from BD Biosciences, BioLegend, or eBioscience (Thermo Fisher Scientific). Unless otherwise indicated, all staining was performed by adding 1:100 dilution of the fluorochrome-conjugated antibody to 0.5 × 10^6^ to 1 × 10^6^ cells and stained at 4°C for 30 to 45 minutes in 100 μL FACS buffer consisting of PBS with 5% FCS. Excess antibody was removed by 2 consecutive washings. All surface staining was performed on ice in staining buffer (2% FCS, 0.1% NaN_3_ in PBS) containing anti-FcR antibodies (2.4G2, Miltenyi Biotec). For intracellular staining, surface markers were stained before fixation/permeabilization of cells (BD Biosciences 554722) followed by dilution of intracellular antibodies and subsequent washes in 10× wash buffer (BD Biosciences 554723). For intranuclear staining, we used transcription buffer set (eBioscience, Thermo Fisher Scientific 00-5521-00) and followed manufacturer’s instructions. For phospho-staining, cells were fixed first by adding dropwise chilled phosflow fix buffer I (BD Biosciences 557870) and kept for 15 minutes on ice. Next, cells were permeabilized with Perm buffer III (BD Biosciences 558051) by adding ice-cold buffer dropwise into the cell pellet on ice and incubated for 20 minutes. Phospho-tagged antibodies were next diluted with PBS according to manufacturer’s recommended dilution. Cells were washed after 30 minutes of incubation and analyzed by flow cytometry. Samples were collected on a FACSCanto II (BD Biosciences) or BD LSRFortessa using FACSDiva software (BD Biosciences), and data were analyzed using FlowJo (Tree Star, Inc.).

### In vitro assays

Depletion of a specific subset of lymphocytes was performed using biotin-labeled antibody followed by exposure to anti-biotin microbeads and magnetic separation (Miltenyi Biotec) according to manufacturer’s protocol. For the human portion of the studies, CD8^+^ T cells were obtained from a combination of sources. For some studies (such as the mass spectroscopy data outlined in Supplemental Figure 1A), CD8^+^ T cells were purchased from StemCell Technologies (catalog 200-0164). For other studies CD8^+^ T cells were either purchased from StemCell Technologies or obtained from healthy volunteers at the University of Maryland under IRB protocol HP-40382. Isolation of untouched CD8^+^ T cells from human PBMCs obtained from healthy volunteers was done by using the human and CD8^+^ T cell isolation kit (catalog 130-096-495). Similarly, mouse CD8^+^ T cell isolation was performed using mouse kit 130-104-075. For plate-bound stimulation of CD8^+^ T cells, we used 10 μg/mL of anti-CD3 (Thermo Fisher Scientific, Anti-Human CD3 [OKT3], functional grade, catalog 16-0037-81; or Anti-Mouse CD3 Monoclonal Antibody [17A2], functional grade, catalog 16-0032-82) and 2 μg/mL of anti-CD28 (Anti-Human CD28 monoclonal antibody [CD28.2], functional grade, catalog 16-0289-81; or CD28 Monoclonal Antibody [37.51], functional grade, catalog 16-0281-82), unless otherwise stated, to coat the plates at 5% CO_2_ at 37°C for 2 hours. Plates were washed with 1× PBS and cells added in RPMI 1640 (with l-glutamine, Gibco, Thermo Fisher Scientific) complete media (10% FBS) containing 1× penicillin-streptomycin (5000 U/mL, Gibco, Thermo Fisher Scientific) for culture. In vitro expansion of T cells isolated from tumor-bearing mice was done in complete media consisting of RPMI 1640 with 10% FBS, 1× penicillin/streptomycin, 50 μM β-mercaptoethanol, and 20 mM l-glutamine. Cells were expanded in vitro with transient anti-CD3/CD28 stimulation (×72 hours) in the presence of continuously replenished IL-2 or OMCPmutIL-2 at a concentration of 5000 IU/mL. For human CD8^+^ T cell expansion, we used AIM-V media (Thermo Fisher Scientific) without serum and supplemented with penicillin-streptomycin. Human T cells were similarly expanded with transient anti-CD3/CD28 stimulation for 72 hours followed by continuously replenished IL-2 or OMCPmutIL-2 at a concentration of 5000 IU/mL.

### Tumor studies

For in vivo tumor studies, we utilized the well-described B16 melanoma model expressing the model tumor antigen ovalbumin (B16^ova^) or Lewis lung carcinoma expressing ovalbumin (LLC^ova^). For both tumor types, we injected 0.5 × 10^6^ of tumor cells subcutaneously into the flank of mice. Tumor growth was tracked through serial measurements of 2 perpendicular diameters and estimated as 4/3πr^3^ for total tumor volume. Tumors were measured 3 times weekly. Once an animal was noted to have a tumor >20 mm in diameter, or to manifest signs of distress or loss of >15% of their body weight, it was sacrificed per IACUC guidelines. For in vivo tumor studies mice received subcutaneous injections of cytokines in 200 μL of saline corresponding to 150,000 IU/d (6 × 10^6^/kg/d).

For tumor growth and survival of LLC^ova^-bearing mice treated with combination of cytokines and checkpoint inhibitors, we injected 0.5 × 10^6^ LLC^ova^ into each mouse and tumor establishment was tracked. After successful implantation of tumor in the flank (minimum 100 cm^3^), we started treatment with a combination of cytokines and checkpoint inhibitors. Cytokines (all cytokines calculated according to molar equivalent described already) were injected 5 days a week (Monday through Friday) adjusted to 150,000 IU/d, subcutaneously, during the entire length of experiment. For checkpoint inhibitors, we used *InVivo*MAb anti-mouse CTLA-4 (CD152) and *InVivo*MAb anti-mouse PD-1 (CD279) (BioXCell BE0131 and BE0146, respectively). We subcutaneously injected 200 μg at 2 doses per week into each mouse receiving the treatment for 4 weeks (or till euthanized). Tumor measurements were done 3 times per week, and experimental end point criteria were followed according to IACUC guidelines as already described.

### Adoptive cell transfer

For analysis of cell persistence, we adoptively transferred 1 × 10^6^ congenic CD45.2 CD8^+^ T cells expanded for 2 weeks either in OMCPmutIL-2, IL-2, or IL-15 (5000 IU/mL) into recipient CD45.1 mouse. For B16 tumor experiment, we adoptively transferred 20 × 10^6^ pmel CD8^+^ T cells expanded in OMCPmutIL-2, IL-2, or IL-15 on day 7 after tumor injection (B16 injected into flanks). All adoptive transfer was done intravenously into the tail vein of mice using a microscope guided technique.

### Seahorse assay

One day prior to the actual experiment, 200 μL/well of distilled water was dispersed in the utility plate. The cartridge sensors (Seahorse mini Fluxpak XFe96, 102601–100, Agilent Technologies) were hydrated, overnight, in a 37°C non-CO_2_ incubator. On the day of the experiment, distilled water was replaced by XF Calibrant (100840–100, Agilent Technologies); cartridge sensors were immersed into the XF Calibrant and incubated for 1 hour in a 37°C non-CO_2_ incubator. A total of 180 μL of XF Calibrant was added in the 4 edge wells in the culture plate. The cover guide was then loaded on the CD8^+^ T cell–seeded culture plate. A total of 20 μL of oligomycin (port A) and 22 μL of FCCP (port B) (from Agilent Seahorse XF Cell Energy Phenotype Test Kit, 103325–100, Agilent Technologies) were then displayed, at a final concentration of 1 μM and 1.5 μM, respectively. The utility plate filled with XF Calibrant and capped with the cartridge was positioned in the Seahorse XFe96 analyzer’s tray (Agilent Technologies), and the calibration of the signals generated by all 96 wells was performed. The culture plate was then introduced in the tray and the acquisition program was started. Data were analyzed through the Wave 2.6.1 Software (Agilent Technologies).

### RNA sequencing and differential gene expression and pathway analysis

#### Sample preparation and RNA extraction.

RNA was extracted from CD8^+^ T cells after different cytokine stimulation according to experimental design, using the TRIzol-based technique according to the manufacturer’s guidelines (Thermo Fisher Scientific).

#### RNA library preparation.

RNA samples were quantified using Qubit 2.0 Fluorometer (Life Technologies, Thermo Fisher Scientific), and RNA integrity was checked with 2100 Bioanalyzer (Agilent Technologies). RNA library preparations, sequencing reactions, and initial bioinformatics analysis were conducted at GENEWIZ, LLC. RNA-Seq data were deposited into the National Center for Biotechnology Information’s Gene Expression Omnibus (GSE202765).

#### Gene expression profiling, pathway analysis, and differential expression.

Analysis was done at the Bioinformatics Core, University of Virginia. Briefly, we collected more than 20 million reads (150 paired-end reads) per sample. RNA-Seq libraries (FASTQ files) were checked for their quality using the FastQC program (http://www.bioinformatics.babraham.ac.uk/projects/fastqc/). The results from FastQC were aggregated using MultiQC software (https://multiqc.info). The contamination of the adaptor sequence was removed by cutadapt (https://cutadapt.readthedocs.io/en/stable/). The clean reads were mapped with the star aligner to the mouse transcriptome (mm10). The HTseq (65) software was used to count aligned reads mapping onto each gene. The count table was imported to R to perform differential gene expression analysis using the DESeq2 package (66). Low expressed genes (genes expressed only in few replicates with low counts) were excluded from the analysis before identifying differentially expressed genes. Data normalization, dispersion estimates, and fitting of a negative binomial model were carried out with the DESeq function. The log-transformed normalized gene expression of the 500 most variable genes was used to perform a principal component analysis. The differentially expressed genes were ranked based on the log_2_ fold change and FDR-corrected *P* values. The ranked file was used to perform pathway analysis. The enriched pathways will be selected based on enrichment scores as well as normalized enrichment scores. The heatmap of selected genes was generated by using p-heatmap package in Bioconductor R.

### Western blot

Isolation of untouched cytotoxic CD8^+^ T cells has been already explained elsewhere in the manuscript. The cells were lysed in situ for 30 minutes at 4°C by the addition of 10× cell lysis buffer containing 20 mM Tris-HCl (pH 7.5), 150 mM NaCl 1 mM Na_2_EDTA, 1 mM EGTA, 1% Triton X-100, and protease inhibitor cocktail. The resulting cell lysates were cleared by centrifugation at 18,800*g*. Antibodies against phospho-Jak1 (clone tyr1034/1035), phospho-JAK3 (clone tyr980/981), pan-JAK1 (catalog 3332), and pan-Jak3 (clone D7B12) were all purchased from Cell Signaling Technology. Antibody against human phospho-NFAT1 (Phospho-Ser54-GTX25246) was purchased from Genetex whereas mouse p-NFAT1 (Ser54) was purchased from Abcam (ab200819). After 3 washes with cell lysis buffer, the proteins were resolved on a 10% Tris-Glycine SDS-PAGE gel (Bio-Rad) and transferred to an Immobilon-P PVDF membrane. Next, the membranes were blocked with 3% BSA in PBS with 0.1% Tween 20 for 1 hour at 20°C. Phosphoproteins were then detected with respective primary antibodies followed by the appropriate secondary antibodies conjugated to horseradish peroxidase. The horseradish peroxidase activity was detected with Pierce enhanced chemiluminescence substrate (Thermo Fisher Scientific) according to the manufacturer’s instructions. The chemiluminescence signal was acquired with the ChemiDoc MP Imaging System and analyzed with the Image Lab 5.1 software application (both from Bio-Rad). Images presented throughout the manuscript have been cropped for presentation.

### Confocal microscopy

Isolation of CD8^+^ T cells was done as previously described in the Methods section.

For staining of lipid rafts and cell surface receptors, we used Alexa Fluor 488–conjugated CTB along with CD3ζ (red dye) and CD122 (far red dye) to perform membrane staining. Cells were stained for the dyes right after completion of specific incubation times in respective cytokines followed by 2 washes in PBS. Cells were fixed and mounted using ProLong Gold Antifade Reagent (Cell Signaling Technology, catalog 8961).

We used MitoTracker Deep Red (Thermo Fisher Scientific) to stain mitochondria of cells. Using DMSO, the MitoTracker probes were diluted to 1 mM. Cells were incubated with 100 nM of MitoTracker Deep Red (excitation 644 nm; emission 665 nm) for 15 minutes. Following incubation, cells were washed 3 times in prewarmed, equilibrated medium.

The images were acquired using a spinning disk confocal (ZEISS LSM 880) microscope equipped with Airyscan 2 in Multiplex mode for efficient super-resolution imaging of a large field of view. *Z*-slices were set to 0.2 micron unless otherwise noted. For high-resolution imaging, we used Plan-Apochromat 40×/1.3, or for confocal modality performance, we used the 63× 1.4NA Plan-Apochromat objective. The 568 nm excitation and 570–615 nm emission filters were used. The confocal images were deconvolved utilizing the constrained iterative deconvolution routines (Zen software version 2.3, ZEISS).

### Electron microscopy

Transmission electron micrographs were recorded at the University of Virginia Molecular Electron Microscopy Core facility (RRID:SCR_019031). We used the Tecnai F20 microscope, which is a field emission 200 kV S/TEM with an X-TWIN objective lens and high brightness field emission electron gun (FEG) optimized for analytical work. Briefly, different culture condition cells were postfixed in 2% osmium tetroxide in Sorensen’s buffer for 1 hour, dehydrated in an ascending ethanol series (30%, 50%, 70%, 90%, 100%), and embedded in Epon/Araldite resin. Samples were stained in a premixed solutions of uranyl acetate and lead citrate and examined at 200 V using an F20 electron microscope having a FEG source of electrons. Negative-stain data collection was implied with a 4k × 4k Ultra Scan charge-coupled device camera running Gatan GIF Quantum with DualEELS and High-Speed Spectrum Imaging.

### Histology

Snap-frozen tumors were embedded in optimal cutting temperature compound (OCT) immediately after harvest. Slides were brought to room temperature and air-dried, then postfixed for 10 minutes in 4% paraformaldehyde, followed by washes in PBS for 5 minutes to remove the OCT. Histological sample preparation and staining were performed by the Research Histology Core and the Biorepository and Tissue Research Facility at the University of Virginia. Slides were imaged using Leica DM6 microscope with Leica DMC6200 camera (Leica Microsystems).

### Inhibitors and peptides

We used FK506 (tacrolimus) for inhibition of NFAT by blocking the calcineurin/NFAT pathway. For all in vitro experiments, we used 100 nM concentration of the inhibitor (Selleckhem). For JAK inhibition, we used Jak1/3 inhibitor (StemCell Technologies) at a concentration of 500 nM for CD8^+^ T cell–related in vitro assays. For Akt inhibition, we used an Akt inhibitor (Santa Cruz Biotechnology sc394003) that is a phosphatidylinositol ether analog that potently and selectively inhibits Akt. All peptides used in the study were a gift from the University of Missouri, Columbia, Missouri, USA, and were used as previously mentioned.

### In vitro cytotoxicity assays

For in vitro lytic assay, we generated CD19-CD28-CD3z CAR-modified human T cells using gamma murine retrovirus transduction. To achieve high-level transduction efficiency, we adequately stimulated T cells with anti-CD3/CD28 beads in the presence of recombinant human IL-2 (40 IU/mL) for 72 hours followed by transduction of activated T cells with retrovirus using retronectin-coated plates, which promotes colocalization of retrovirus with target cells to dramatically enhance transduction efficiency. The transduced T cells were expanded in respective cytokines (OMCPmutIL-2, IL-2, or IL-15) for 3 more days, in AIM-V (300 IU/mL of respective cytokines). The level and frequency of CAR expression in transduced T cells was determined by the ability of protein L to bind to all classes of immunoglobulin that contain kappa light chains, including single chain variable fragments as used in CAR T cells. Biotinylated protein L was then detected through flow cytometry by staining cells with fluorophore-conjugated streptavidin. In addition to work performed internally in our laboratory, similar CD19-FLAG-CD28-CD3z CARs were generated by ProMab Biotechnologies Inc. on a fee-for-service basis according to established protocols with validation of growth and killing experiments.

Nalm6 is a B cell precursor leukemia cell line that was used as a target cell, and cytotoxicity of effector CAR T cells was calculated as a direct index of lysis of the CD19^+^ NALM6 cell line (ATCC CRL-3273) by CAR T cells expanded in OMCPmutIL-2, IL-2, or IL-15. We used effector/target ratio of 5:1; 2.5:1, 1:1, and 1:2 with 100,000 target cells (Nalm6) and adjusted the number of effector cells according to ratio. Cells were incubated overnight (16 hours), and flow cytometric analysis was done using fixable live/dead staining (Invitrogen, Thermo Fisher Scientific) to quantify CAR T cell–mediated in vitro tumor lysis.

The in vitro cytotoxicity assay involving B16 melanoma lysis by syngeneic CD8^+^ T cells was done by exposure of tumor cells (target) to purified CD8^+^ T cells (effectors) for 18 hours with effector/target ratio of 1:1. Next cells were washed and labeled with anti-CD8 antibody and analyzed for apoptosis/lysis using fixable live/dead stains (Thermo Fisher Scientific L34959). Cells were gated as CD8^+^ T cells and B16 melanoma based on CD8^+^ staining and then analyzed for viability using flow cytometric method.

### Statistics

All statistics were performed using Prism (GraphPad) software. For most assays, a 2-tailed *t* test was used for 2 comparisons, and 1-way ANOVA was used for multiple comparisons, as indicated in the appropriate figure legends. For survival curves, comparison was performed by log-rank (Mantel-Cox) test. For relative ratio of MFI, data were compared to the null hypothesis of 1. Data in figures are presented as mean ± SEM. A *P* value of more than 0.05 is assumed to be not statistically significant.

For modeling domain size with line tension and leaflet dipoles, we used the model of Usery et al. (35) to provide insight into how raft size is influenced by an increase in line tension due to the fusion protein cytokine, OMCPmutIL-2, being located and increasing the stability of the raft domains. In this model, the total energy of the raft/disordered σ domain bilayer is the sum of the energy from the perimeter of the 2 domains (*E_perim_*) and the electrostatic potential energy (*E_elec_*) that is modeled from permanent lipid dipoles of each leaflet. The minimum of the total energy (*E_total_*) as a function of domain radius (*R_D_*) determines the raft size:

 (Equation 1)
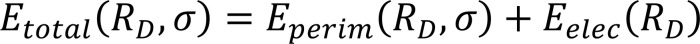


where the perimeter energy depends on how many domains (*N_D_*) and the line tension (σ):

 (Equation 2)
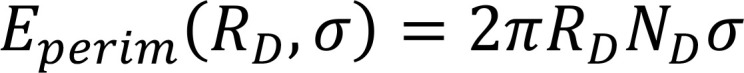


The number of domains is estimated from a single total area covered from all domains as 20 μm and thus is:

 (Equation 3)
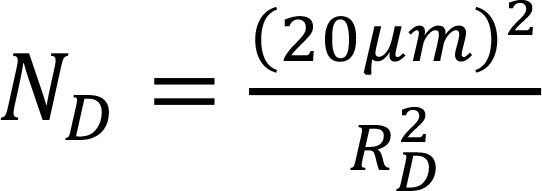


It is assumed that the lipids are distributed identically in the 2 bilayer leaflets and the bilayer normal is the dipole alignment reference yielding an energy contribution from the intra- and interleaflet:

 (Equation 4)
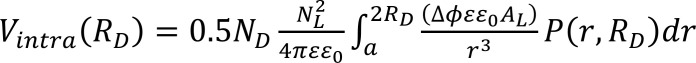


 (Equation 5)
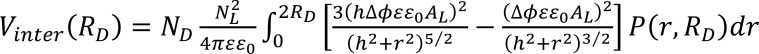


where the area per lipid (*A_L_*) is assumed to be 55 Å (38) and the separation of distance between opposing dipoles, *h* = 3.5 nm (taken as the average of the hydrocarbon thickness, *D_C_*, and bilayer thickness, *D_B_*, to represent the carbonyl-glycerol position) based on simulations of raft-like domains with sphingolipids (38) and GM1 (39). Other terms in the equation are the permittivity of free space, ε*_0_*; dielectric constant of the bilayer near the dipoles, ε = 8; distance of closest approach of the 2 dipoles, ; and the electrostatic potential difference between the raft domain and surrounding disordered phase, . The integrals in equations 4 and 5 are assuming a probability distribution of dipole separations distance given as a pair distance distribution function for a disk normalized with units of *r* in nm:
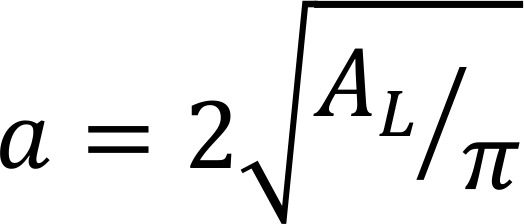

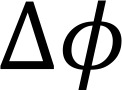


 (Equation 6)
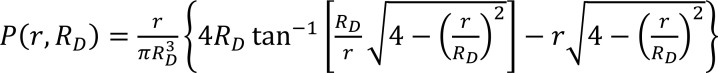


Then the electrostatic potential energy becomes:

 (Equation 7)
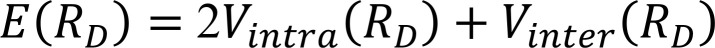


### Study approval

All protocols were approved by the corresponding IRBs (University of Virginia and University of Maryland). The Animal Care and Use Committees (University of Virginia and University of Maryland) approved all procedures performed in this study, and all the procedures adhered to the guidelines outlined in the NIH *Guide for the Care and Use of Laboratory Animals* (National Academies Press, 2011). All studies met the ethical and humane criteria for transportation, housing, and care established by NIH guidelines.

## Author contributions

AB, AS, ERL, and ASK designed research; AB, DL, YG, ZM, and KC performed experiments; JBK, AB, and ASK analyzed data; AB, ASK, and ERL wrote the manuscript; and CL and JW provided feedback on the manuscript.

## Supplementary Material

Supplemental data

## Version 1 05/23/2022

Electronic publication
