# Supplementary material for: A reengineered common chain cytokine augments CD8+ T cell–dependent immunotherapy
Source: JCI Insight. 2022 May 23;7(10):e158889. doi: 10.1172/jci.insight.158889 (PMC9220948; doi:10.1172/jci.insight.158889)
Supplement: Supplemental data [file jciinsight-7-158889-s192.pdf]

A Re-Engineered Common γ Chain Cytokine

Supplemental Figures:

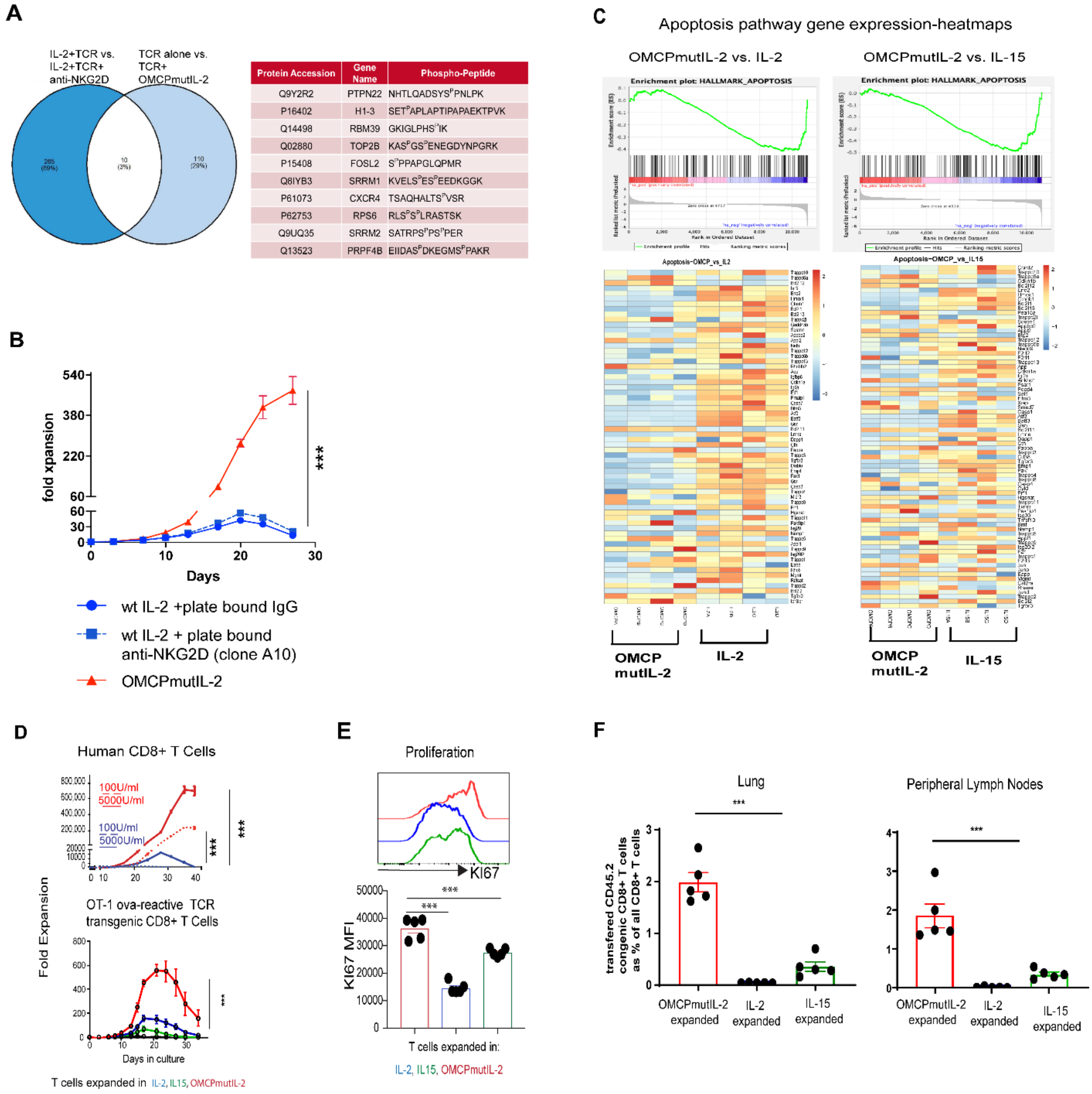

Protein Accession	Gene Name	Phospho-Peptide
Q9Y2R2	PTPN22	NHTLQADSYS^{P}PNLPK
P16402	H1-3	SET^{P}APLAPTIPAPAEKTPVK
Q14498	RBM39	GKIGLPHS^{P}IK
Q02880	TOP2B	KAS^{P}GS^{P}ENEGDYNPGRK
P15408	FOSL2	S^{P}PPAPGLQPMR
Q8IYB3	SRRM1	KVELS^{P}ES^{P}EEDKGGK
P61073	CXCR4	TSAQHALTS^{P}VSR
P62753	RPS6	RLS^{P}S^{P}LRASTSK
Q9UQ35	SRRM2	SATRPS^{P}PS^{P}PER
Q13523	PRPF4B	EIIDAS^{P}DKEGMS^{P}PAKR

Supplemental Figure 1 (for high resolution tiff please see: https://figshare.com/s/65539ec488d6029b1053) **: (A)** In order to further expand on already

published data (Figure 6 of (17)) demonstrating that OMCPmutIL-2 does not contribute to canonical NKG2D signal transduction we turned to phosphoprotein analysis by mass spectroscopy. Human CD8+ T cells were stimulated through their TCR in the presence of anti-CD3/28 agonistic antibodies in the presence of IL-2 or IL-15 in addition to plate-bound NKG2D crosslinking agonistic antibodies. A total difference in 275 phospho-peptide defined genes was identified between these two groups. In parallel human CD8+ T cells were stimulated through their TCR in the presence of plain media or media containing OMCPmutIL-2. A total difference in 120 phospho-peptide defined genes was identified between these two groups. Nevertheless only 10 phospho-peptide defined genes (3% of total proteins) were shared between these conditions. Such data further supports our hypothesis that OMCPmutIL-2 does not activate canonical NKG2D-defined signaling pathways in CD8+ T cells. (**B**) Expansion of murine CD8+ T cells with plate bound anti CD3/28 in the presence of IL-2+IgG, IL-2+ antiNKG2D and OMCPmutIL-2 revealed that the simple addition of NKG2D crosslinking did not lead to a substantial expansion of murine CD8+ T cells that could rival OMCPmutIL-2-driven expansion. (**C**) Analysis of differential expression of genes involved in apoptosis pathway as expressed by enrichment scores(top) and heatmaps(bottom). **(D)**Expansion of human peripheral blood-derived CD8+ T cells stimulated by plate bound anti-CD3/CD28 in the presence of low (100U/ml) or high (5000U/ml) IL-2 or OMCPmutIL-2 (top) and OT-1 anti-ovalbumin TCR transgenic CD8+ T cells in co-culture with dendritic cells from C57BL/6-Tg(CAG-OVAL)916Jen/J mice that express membrane bound chicken ovalbumin with either no additional cytokine (black), wild-type IL-2 (blue), IL-15 (green) or OMCPmutIL-2 (red) at 5000U/ml. (**E**) KI67 expression in naïve CD8+ T cells stimulated through their TCR in the presence of IL-2, IL-15, or OMCPmutIL-2. Representative FACS plot(top) and MFI quantification (bottom) of KI67 expression shown here. (**F**) Analysis of lung

and peripheral lymph nodes, 50 days after adoptive transfer of CD45.2 congenic CD8+ T cells expanded in wild-type IL-2 (blue), IL-15 (green) or OMCPmutIL-2 (red) prior to transfer. ns-p >.05; *p<.05; **p<.01; ***p<.001

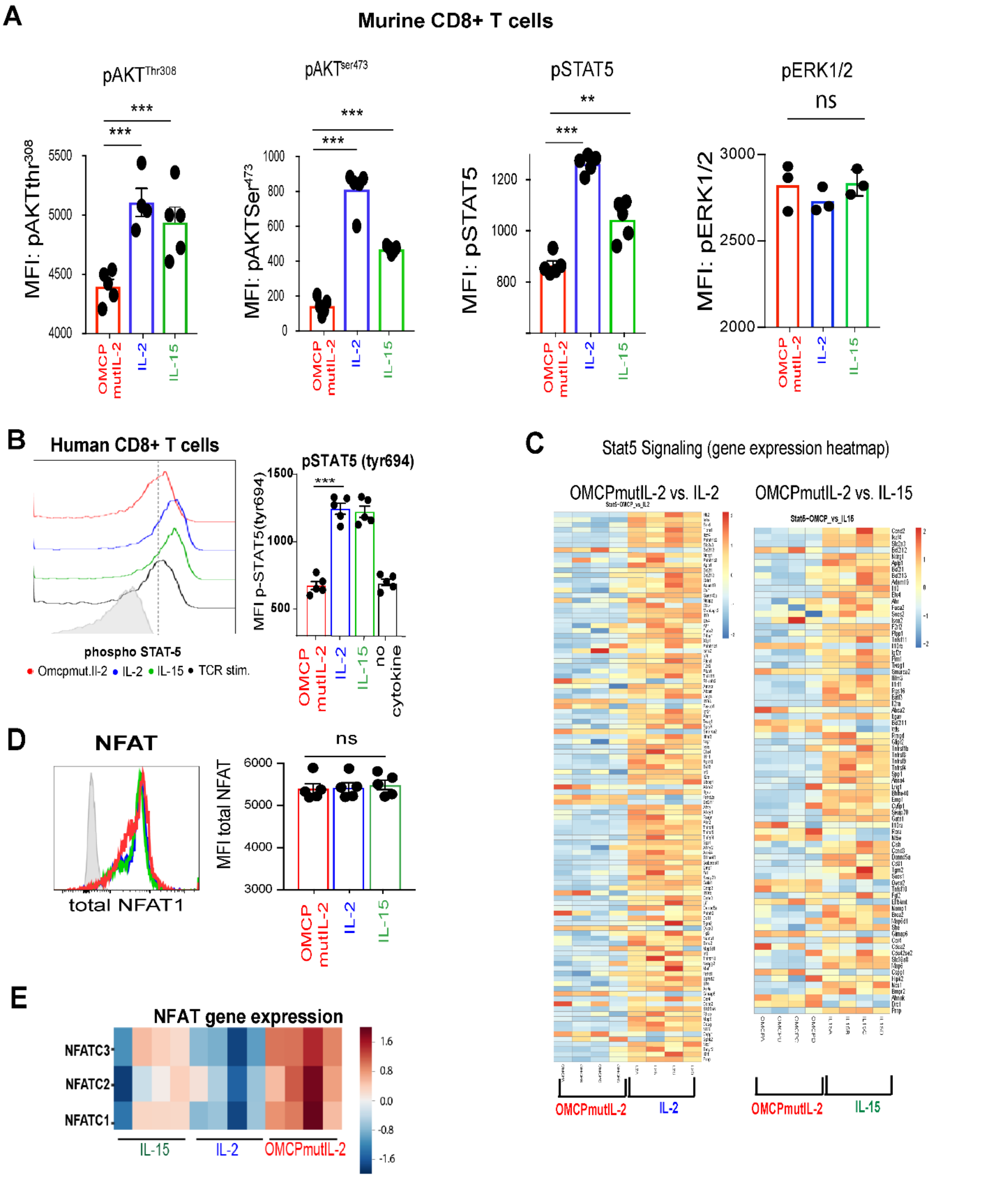

Supplemental Figure 2: (for high resolution tiff please see: https://figshare.com/s/1cdc0e591b3ebc75bb34 **) : (A)** Flow cytometric analysis of phospho-Akt,

phosphor-STAT-5 and phospho- ERK after in vitro activation of murine CD8+ T cells. **(B)** Median fluorescence intensity (MFI) of phospho-STAT5 in human CD8+ T cells after stimulation with OMCPmut.IL-2(red), IL-2(blue), IL-15 (green) and TCR stimulation alone (black) with isotype (solid grey) as graphic representative histograms (left panel) of a mean of 5 individual samples (right panel). **(C)** Analysis of differential expression of genes involved STAT5- signaling in CD8+ T cells expanded in the presence of IL-2, IL-15 vs OMCPmutIL-2. **(D)** Total NFAT levels in splenic CD8+ T cells expanded in IL-2, IL-15 or OMCPmutIL-2. **(E)** NFAT gene expression by quantitative RNA sequencing ns-p >.05; *p<.05; **p<.01; ***p<.001

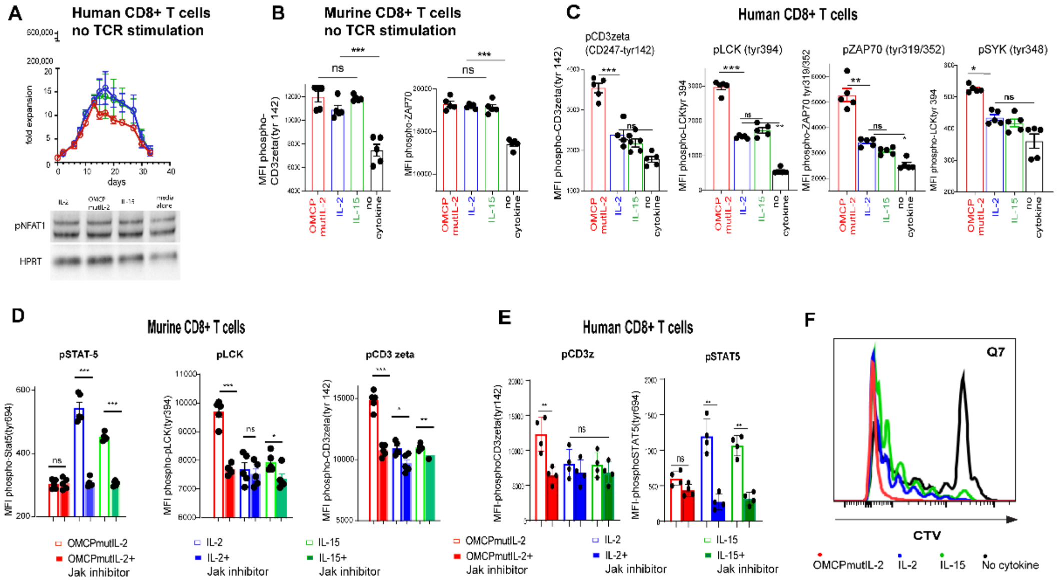

Supplemental Figure 3: (for high resolution tiff please see: https://figshare.com/s/15404a5c8524ae461217) **: (A)** Expansion of human CD8+ T cells and NFAT phosphorylation in the presence of 5000IU/ml of various cytokines without TCR crosslinking. **(B)** PhosphoCD3ζ and Zap70 phosphorylation in the presence of cytokines but no TCR activation. **(C)** Phosphorylation of TCR signaling components of human CD8+ T cells in the presence of wild-type IL-2 (blue), IL-15 (green) or OMCPmutIL-2 (red) with TCR agonistic antibodies (stimulation with 10 ug/ml anti-CD3 and 2ug/ml anti CD28). **(D)** PhosphoCD3ζ and phosphorSTAT5 levels in wild-type murine and **(E)** Human CD8+ T cells in the presence or absence of Jak1/3 inhibitor. **(F)** Proliferation of murine OT-1 CD8+ T cells, as measured by diminution of the proliferation dye Cell Trace Violet (CTV), when cultured for 7 days in the presence of Q7 peptide loaded dendritic cells and various cytokines. ns-p >.05; *p<.05; **p<.01; ***p<.001

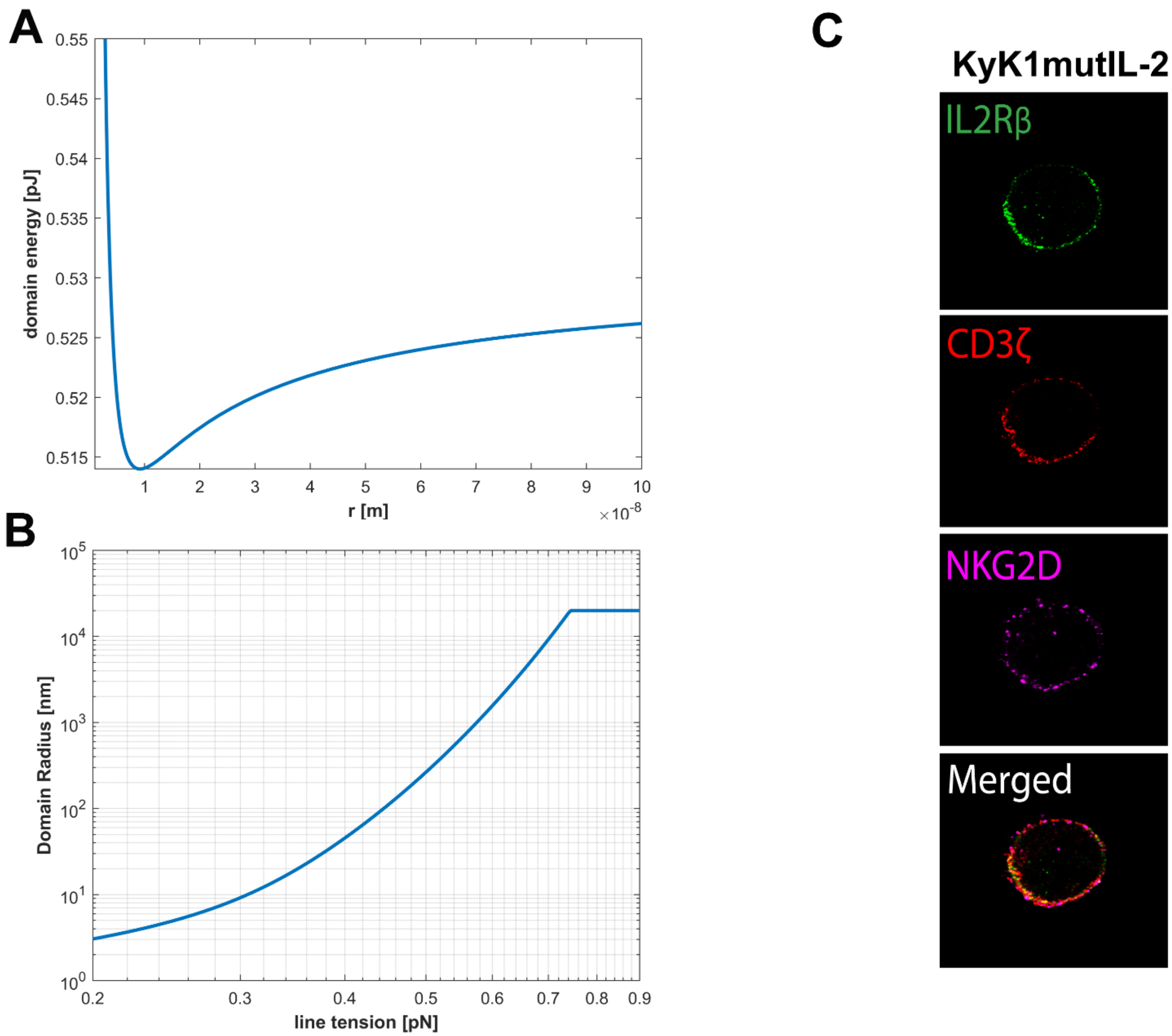

Supplemental Figure 4 (for high resolution tiff please see: https://figshare.com/s/4dd480616d077b3d6b89 **): (A)** The E-total as a function of RD with a σ=0.3pN. **(B)** Raft domain size as a function of line tension with a value of 20μm as the limit of a single domain based on the model total area of raft domains described above. As shown in Supplemental Figure A, for a line tension of 0.3pN, the minimum in the energy is at 9.5 nm. This plot was done for all reasonable values of line tension to fine the domain size given a line tension. In this log-log plot, there is a stronger than exponential increase in domain radius as the line tension

increases. **(C)** Confocal images of receptor localization of human CD8+ T cell surface receptors IL-2Rβ(CD122) (green) CD3ζ (red) and NKG2D (pink) after one hour in presence of plate-bound anti-CD3/28 and stimulated with kyk1mut.IL-2(lower NKG2D affinity compared to OMCPmutIL-2) cytokine

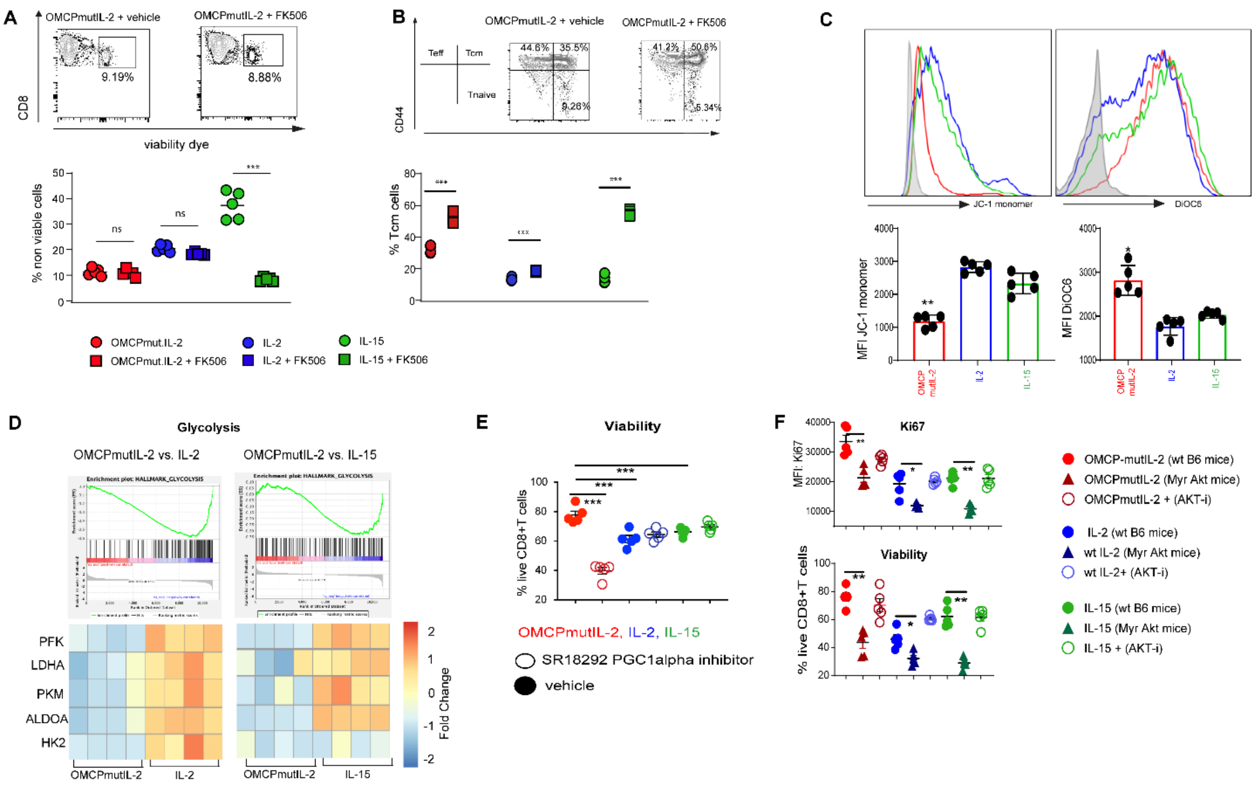

Supplemental Figure 5: (for high resolution tiff please see: https://figshare.com/s/4dd480616d077b3d6b89) CD8+ T cell viability **(A)** and CD44^{high}CD62L^{high} central memory cell (Tcm) generation **(B)** in the presence of absence of NFAT inhibition with FK506. **(C)** Evaluation of JC-1 monomer (J aggregates and measure of mitochondrial depolarization) and DiOC6 as an index of mitochondrial membrane potential **(D)** Analysis of differential expression of genes involved in glycolysis as expressed by enrichment scores and heatmaps. **(E)**Viability of CD8+ T cells in the presence or absence of PGC1α inhibitor. **(F)** Proliferation as measured by KI67 expression, and viability of murine CD8+ T cells in the presence of constitutively active AKT (myrAKT), or AKT inhibition. ns-p >.05; *p<.05; **p<.01; ***p<.001

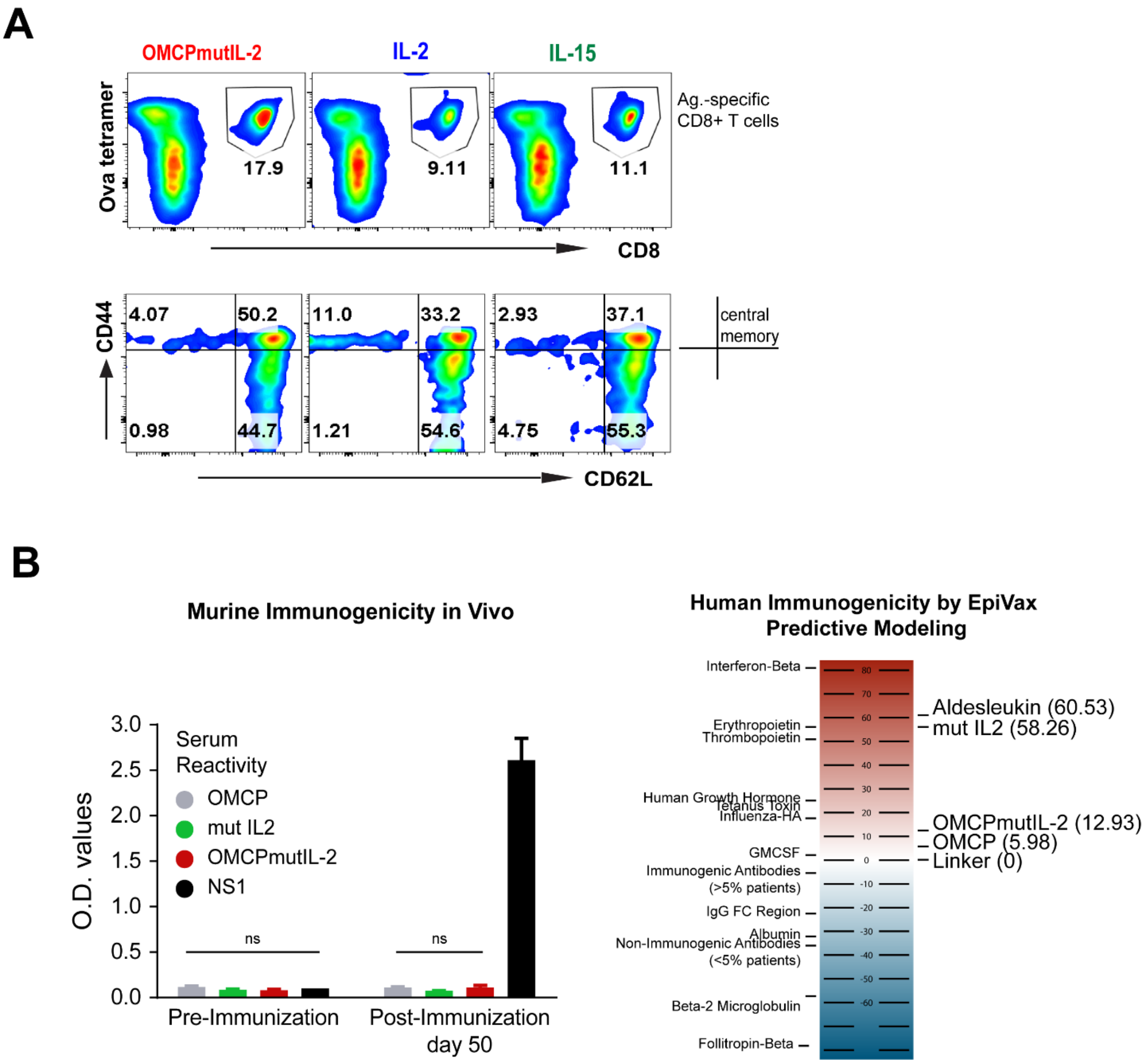

Supplemental Figure 6: (for high resolution tiff please see: https://figshare.com/s/f6b9a6b69b1dcfceceb9) **(A)** Circulating antigen specific CD8+ T cells and central memory CD8+ T cells after two weeks of cytokine administration in mice with established LLC^{ova} flank tumors. (**B-left** panel) Serum antibody reactivity to OMCPmutIL-2 as well as its various components pre and 50 days post one 5 days cycle of 750,000IU. West Nile virus NS-1 protein is a positive control for immunogenicity. Data representative of 5 mice per group. (**B-right**

panel) Predictive immunogenicity modeling of OMCPmutIL-2 compared to Aldesleukin (IL-2 in current clinical use). Predicted immunogenicity of commonly used drugs on the left side of the panel. Higher score = more immunogenic. ns-p >.05.
